# Supplementary figures and images for: Effectiveness of a telenursing intervention program in reducing exacerbations in patients with chronic respiratory failure receiving noninvasive positive pressure ventilation: A randomized controlled trial
Source: PLoS One. 2023 Oct 26;18(10):e0269753. doi: 10.1371/journal.pone.0269753 (PMC10602241; doi:10.1371/journal.pone.0269753)

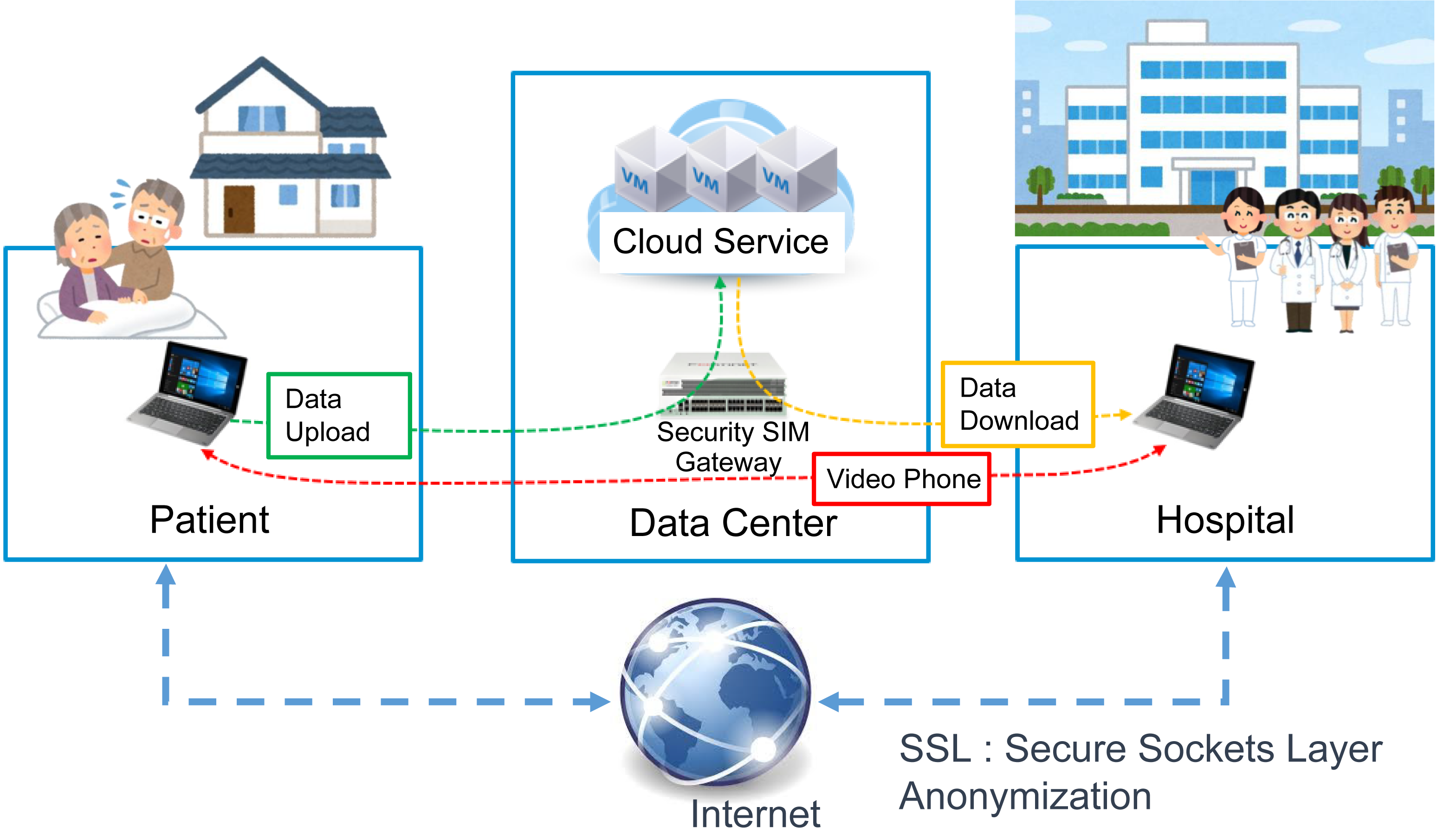

Supplement: S1 Appendix — (TIF) [file pone.0269753.s002.tif]

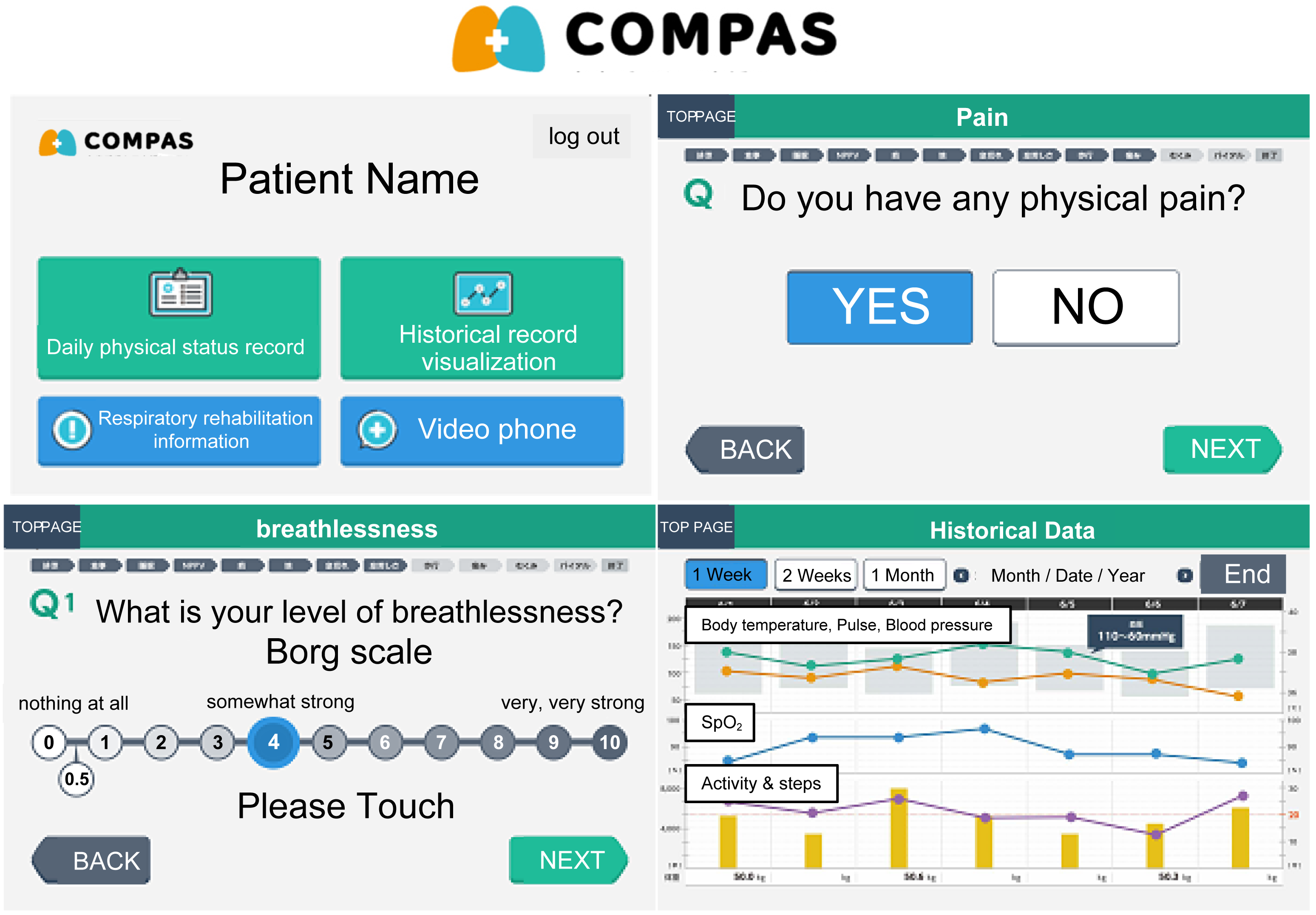

Supplement: S2 Appendix — (TIF) [file pone.0269753.s003.tif]
