# Supplementary material for: Effectiveness of a telenursing intervention program in reducing exacerbations in patients with chronic respiratory failure receiving noninvasive positive pressure ventilation: A randomized controlled trial
Source: PLoS One. 2023 Oct 26;18(10):e0269753. doi: 10.1371/journal.pone.0269753 (PMC10602241; doi:10.1371/journal.pone.0269753)
Supplement: S5 Appendix — (DOCX) [file pone.0269753.s006.docx]

| **S5 Appendix**  Differences between baseline and 3 months post-enrollment for Primary endpoint | | | | |
| --- | --- | --- | --- | --- |
|  | Intervention group(n=15) | |  |  |
|  |  | Number of hospitalizations in the past 3 months (times) | Number of days hospitalized in the past 3 months (days) | Number of unscheduled outpatient visits in the past 3 months (times) |
| ID |  |  |  |  |
|  | A001 | 0 | 0 | 1 |
|  | A002 | 0 | 0 | 0 |
|  | A003 | 0 | 0 | 0 |
|  | A004 | 0 | 0 | 0 |
|  | A005 | 0 | 0 | 0 |
|  | A006 | 0 | 0 | 2 |
|  | A007 | 0 | 0 | 0 |
|  | A008 | 0 | 0 | 0 |
|  | A009 | 0 | 0 | 0 |
|  | A010 | -1 | -30 | 1 |
|  | A011 | 0 | 0 | 2 |
|  | A012 | 0 | 0 | 0 |
|  | A013 | -1 | -20 | 0 |
|  | A014 | 0 | 0 | 1 |
|  | A015 | -1 | -30 | 0 |
| Statistics | |  |  |  |
|  | mean | -0.20 | -5.33 | 0.47 |
|  | Standard deviation | 0.41 | 11.25 | 0.74 |
|  | Median | 0.0 | 0.0 | 0.0 |
|  | Interquartile Range | 0.0 - 0.0 | 0.0 - 0.0 | 0.0 - 1.0 |
|  |  |  |  |  |
|  |  |  |  |  |
|  | Control group(n=16) | |  |  |
|  |  | Number of hospitalizations in the past 3 months (times) | Number of days hospitalized in the past 3 months (days) | Number of unscheduled outpatient visits in the past 3 months (times) |
| ID | |  |  |  |
|  | B001 | 0 | 0 | -1 |
|  | B002 | 1 | 10 | 1 |
|  | B003 | 0 | 0 | 0 |
|  | B004 | 0 | 0 | 0 |
|  | B005 | 0 | 0 | 0 |
|  | B006 | 1 | 12 | -2 |
|  | B007 | 0 | 0 | 0 |
|  | B008 | 0 | 0 | 1 |
|  | B009 | 0 | 0 | -1 |
|  | B010 | 0 | 0 | 0 |
|  | B011 | 0 | 0 | 0 |
|  | B012 | -1 | -7 | 0 |
|  | B013 | 0 | 0 | 0 |
|  | B014 | 1 | 14 | 0 |
|  | B015 | 0 | 0 | 0 |
|  | B016 | 1 | 14 | 0 |
| Statistics | |  |  |  |
|  | mean | 0.19 | 2.69 | -0.13 |
|  | Standard deviation | 0.54 | 6.16 | 0.72 |
|  | Median | 0.0 | 0.0 | 0.0 |
|  | Interquartile Range | 0.0 - 0.8 | 0.0 - 7.5 | 0.0 - 0.0 |
