# Supplementary material for: Effectiveness of a telenursing intervention program in reducing exacerbations in patients with chronic respiratory failure receiving noninvasive positive pressure ventilation: A randomized controlled trial
Source: PLoS One. 2023 Oct 26;18(10):e0269753. doi: 10.1371/journal.pone.0269753 (PMC10602241; doi:10.1371/journal.pone.0269753)
Supplement: S1 File — (DOCX) [file pone.0269753.s007.docx]

6th edition, January 10, 2017

| **Type III**  Effectiveness of a tele-nursing intervention program to prevent acute exacerbations in patients with chronic respiratory failure receiving non-invasive positive-pressure ventilation  (Clinical Trial Registration Number：UMIN000027657)  Principal Investigator  Professor Fumiko Sato  Department of Cancer Nursing, Division of Family Support Nursing, Department of Health Sciences, Tohoku University Graduate School of Medicine  2-1 Seiryo-cho, Aoba-ku, Sendai-shi, Miyagi 980-8575  Tel: 022−717−7926 Fax: 022−717−7910  E-mail:　fsato@med.tohoku.ac.jp  Research Secretariat  Doctoral Program: Makoto Shimoyama  Department of Cancer Nursing, Division of Family Support Nursing, Department of Health Sciences, Tohoku University Graduate School of Medicine  2-1 Seiryo-cho, Aoba-ku, Sendai-shi, Miyagi 980-8575  Tel: 090−7798−1240  E-mail:　makoto.shimoyama.r7@dc.tohoku.ac.jp  July 21, 2017 Created (6th Edition) |
| --- |

Table of Contents

[0. Overview 4](#_Toc104322383)

[1. Objective 7](#_Toc104322384)

[2. Background and rationale for the research plan 7](#_Toc104322385)

[2.1. Background 7](#_Toc104322386)

[2.2. Scientific rationale of the study 13](#_Toc104322387)

[3. Selection policy for research participants 14](#_Toc104322388)

[3.1. Eligibility criteria 14](#_Toc104322389)

[3.2. Exclusion criteria 15](#_Toc104322390)

[4. Methods and duration of the study 15](#_Toc104322391)

[4.1. Research design 15](#_Toc104322392)

[4.2. Overview of the distance nursing intervention program and observations 16](#_Toc104322393)

[4.3. Combined therapy 19](#_Toc104322394)

[4.4. Survey Schedule 20](#_Toc104322395)

[4.5. Research period 21](#_Toc104322396)

[5. Adverse event assessment 21](#_Toc104322397)

[5.1. Obtaining information 21](#_Toc104322398)

[5.2. Description of adverse events 24](#_Toc104322399)

[6. Response to serious adverse events/failures (including the scope of adverse events to be reported to the head of the research institution) 24](#_Toc104322400)

[6.1. Response to adverse events/failures 24](#_Toc104322401)

[6.2. Report to the head of the research institution and the principal investigator 25](#_Toc104322402)

[6.3. Reporting to collaborating institutions 26](#_Toc104322403)

[7. Evaluation items and methods 27](#_Toc104322404)

[7.1. Primary endpoints 27](#_Toc104322405)

[7.2. Secondary endpoints 27](#_Toc104322406)

[7.3. Centralized evaluation system 28](#_Toc104322407)

[8. Registration and allocation 28](#_Toc104322408)

[8.1. Registration 28](#_Toc104322409)

[8.2. Allocation 29](#_Toc104322410)

[9. Number of planned cases and basis for setting 29](#_Toc104322411)

[9.1. Planned number of cases 29](#_Toc104322412)

[9.2. Basis for setting 29](#_Toc104322413)

[10. Statistical analyses 30](#_Toc104322414)

[10.1. Statistical analysis methods 30](#_Toc104322415)

[10.2. Interim analysis and early termination of studies 30](#_Toc104322416)

[11. Methods of data management and self-inspection 31](#_Toc104322417)

[11.1. CRF preparation 31](#_Toc104322418)

[11.2. CRF self-inspection 31](#_Toc104322419)

[11.3. Sending and storage of CRFs 32](#_Toc104322420)

[11.4. CRF correction procedure 32](#_Toc104322421)

[12. Procedures for obtaining informed consent 33](#_Toc104322422)

[12.1. Explanation to research participants 33](#_Toc104322423)

[12.2. Agreement 34](#_Toc104322424)

[13. Procedures for obtaining consent by proxy 35](#_Toc104322425)

[14. Handling of personal information 35](#_Toc104322426)

[14.1. Purpose of use of personal information 35](#_Toc104322427)

[14.2. Method of use of personal information (method of anonymization) 35](#_Toc104322428)

[14.3. Safety management responsibility system (safety management measures for personal information) 36](#_Toc104322429)

[15. Burden on research participants, anticipated risks (including possible adverse events) and benefits, a comprehensive assessment of these risks, and measures to minimize the burden and risks 36](#_Toc104322430)

[15.1. Summary of anticipated benefits and disadvantages associated with participation in the study 37](#_Toc104322431)

[16. Methods of storage and disposal of samples and information 38](#_Toc104322432)

[16.1. Storage of samples and information 38](#_Toc104322433)

[16.2. Disposal of samples and information 38](#_Toc104322434)

[17. Sources of funding for the research and research institution and researcher conflicts of interest 38](#_Toc104322435)

[18. Intellectual property 39](#_Toc104322436)

[19. Methods of disclosing research-related information 39](#_Toc104322437)

[19.1. Registration of the research plan 39](#_Toc104322438)

[19.2. Registration of research results 39](#_Toc104322439)

[19.3. Publication of research results 40](#_Toc104322440)

[20. Report content and method of reporting to the head of the research institution 40](#_Toc104322441)

[21. Responding to consultations from research participants and their concerned parties 40](#_Toc104322442)

[22. A detailed statement on any financial burden or honorarium to the research participants 41](#_Toc104322443)

[23. A statement on whether or not compensation is provided for research-related health hazards and the nature of such compensation for research involving invasive procedures 41](#_Toc104322444)

[24. A statement describing the possibility that the sample/information may be used for future research or provided to other research institutions 42](#_Toc104322445)

[25. Provision of samples and information to collaborating research institutions of this research or other research institutions 42](#_Toc104322446)

[26. Provision of samples and information to commercial entities 42](#_Toc104322447)

[27. Changes to the research protocol 43](#_Toc104322448)

[28. Research implementation structure 44](#_Toc104322449)

[28.1. Name of the research institution and principal investigator 44](#_Toc104322450)

[28.2. Joint research institutions 44](#_Toc104322451)

[28.3. Role of the research secretariat and researchers 46](#_Toc104322452)

[28.4. Statistical analysis and data center 47](#_Toc104322453)

[28.5. Contact for research inquiries 48](#_Toc104322454)

[29. References 49](#_Toc104322455)

[30. Appendix 54](#_Toc104322456)

# Overview

- 1. **Schema**

|  | Patients with chronic respiratory failure receiving NPPV during outpatient visits | | | | | | | |  |
| --- | --- | --- | --- | --- | --- | --- | --- | --- | --- |
|  |  |  |  |  |  |  |  |  |  |
|  | During a routine outpatient visit, the attending physician or physician in charge will provide an overview of the study, to seek for patients who are willing to participate in the research | | | | | | | |  |
|  |  |  |  |  |  |  |  |  |  |
|  |  | Researcher explains the research overview, consenting participants fills out the consent form and are registered | | | | | |  |  |
|  |  |  |  |  |  |  |  |  |  |
|  | At enrollment (baseline)  Primary endpoint: Number of hospitalizations, number of hospital days, and number of unscheduled outpatient visits in the past 3 months  Secondary endpoints: SGRQ score, EQ-5D index, SCAQ responses, respiratory function test, and 6-min walk test | | | | | | | |  |
|  |  |  |  |  |  |  |  |  |  |
|  |  |  |  |  |  |  |  |  |  |
|  |  |  |  |  |  |  |  |  |  |
|  |  | Allocation (allocation adjustment factor: facility) | | | | | |  |  |
|  |  |  |  |  |  |  |  |  |  |
|  |  |  |  |  |  |  |  |  |  |
| Intervention group:  Usual outpatient care + tele-nursing intervention program | | | | |  | Control group:  Usual outpatient care | | | |
|  |  |  |  |  |  |  |  |  |  |
|  |  |  |  |  |  |  |  |  |  |
| Regular outpatient care (once/month)   1. Medical treatment by a physician 2. Health consultation and information by nurses   Remote nursing intervention program (1 time/day, 3 months)  Remote monitoring  Once a day  Observation of information input to the tablet  Input items: Vital signs, respiratory symptoms, food intake, excretion, medication, and physical symptoms other than respiratory symptoms  2. Health counseling and information provision  Health consultation and information provision via videophone once a day for approximately 30 min when triggers are applicable or when the participant wishes | | | | |  | Regular outpatient care (once/month)   1. Medical treatment by a physician 2. Health consultation and information by nurses | | | |
|  |  |  |  |  |  |  |  |  |  |
|  |  |  |  |  |  |  |  |  |  |
|  |  |  |  |  |  |  |  |  |  |
|  |  |  |  |  |  |  |  |  |  |
|  |  |  |  |  |  |  |  |  |  |
|  |  |  |  |  |  |  |  |  |  |
|  |  |  |  |  |  |  |  |  |  |
|  |  |  |  |  |  |  |  |  |  |
|  |  |  |  |  |  |  |  |  |  |
|  |  |  |  |  |  |  |  |  |  |
|  |  |  |  |  |  |  |  |  |  |
|  |  |  |  |  |  |  |  |  |  |
|  |  |  |  |  |  |  |  |  |  |
|  |  |  |  |  |  |  |  |  |  |
|  |  |  |  |  |  |  |  |  |  |
|  |  |  |  |  |  |  |  |  |  |
|  | [Three months after enrollment]  Primary endpoint: Number of hospitalizations, number of hospital days, and number of unscheduled outpatient visits in the past 3 months  Secondary endpoints: SGRQ score, EQ-5D index, SCAQ responses, respiratory function test, and 6-min walk test | | | | | | | |  |
|  |  |  |  |  |  |  |  |  |  |
|  |  |  |  |  |  |  |  |  |  |
|  |  |  |  |  |  |  |  |  |  |
|  | [Expected effects of the intervention]  The tele-nursing intervention program is expected to ameliorate the self-management behavior of the intervention group and to significantly improve the primary and secondary endpoints compared with the control group | | | | | | | |  |
|  |  |  |  |  |  |  |  |  |  |
|  |  |  |  |  |  |  |  |  |  |

- 1. **Objective**

The purpose of this randomized, controlled trial is to evaluate the effect of a tele-nursing intervention program utilizing Information and Communication Technology (ICT) on the prevention of acute exacerbations in patients with chronic respiratory failure receiving noninvasive positive-pressure ventilation (NPPV) in home care.

- 1. **Participants**

We included patients aged at least 20 years, with chronic respiratory failure, receiving NPPV, attending the outpatient Department of Respiratory Medicine at Tohoku University Hospital and cooperating institutions, without cognitive impairment, and who provided informed consent for study participation.

- 1. **Number of planned cases and study period**

(1) Number of planned cases

Based on previous literature, the average number of visits to the emergency department due to acute exacerbation of chronic respiratory disease in patients who use and do not use NPPV is 2.4 and 1.6 times/year, respectively; hence, we assume that the number of visits will improve by 1.0 time/year. The significance level will be 5%, the power will be 80%, and the standard deviation of the number of visits assumed to reduce the occurrence of acute exacerbations will be 1.0 visit/year. In addition, the proportion of included patients who drop out in the middle of the study is assumed to be 10% of the total number of patients; in addition, the intervention and control groups will comprise 17 patients each. Therefore, the planned number of cases for this study is 34.

(2) Research period

After Ethics Committee approval (May 2017) - March 2019

(3) Registration period

After Ethics Committee approval - October 2018

- 1. **Contact information**

(1) Eligibility criteria, treatment modification criteria, and other criteria requiring clinical judgment, registration procedures, and case record form (CRF) entry

Research Secretariat

Doctoral Program: Makoto Shimoyama

Department of Cancer Nursing, Division of Family Support Nursing, Department of Health Sciences, Tohoku University Graduate School of Medicine

Tel: 090−7798−1240

E-mail:　makoto.shimoyama.r7@dc.tohoku.ac.jp

# Objective

The effectiveness of an ICT-based tele-nursing intervention program for patients with chronic respiratory failure undergoing NPPV in home care will be examined in terms of the number of readmissions and occasional visits due to acute exacerbations or chronic respiratory diseases, with readmission and occasional visit rates as primary endpoints.

# Background and rationale for the research plan

# Background

In recent years, Japan's population has been aging at a rate unparalleled in other countries, and by 2025, when the baby boom generation will be aged at least 75 years, there would be a need to establish a community-based comprehensive care system that will enable people to continue living their lives in their familiar neighborhoods until death, even if they require nursing care [1-3]. In addition, approximately 300,000 beds are planned to be transferred from hospitals to home care centers by 2025 [4], and the shift from hospital-based to community-based care is being accelerated [5]. Telemedicine utilizing ICT is a means to support the operation of the community-based comprehensive care system [6]. In Japan, ICT-based telemedicine is considered to contribute greatly to the creation of new wealth and efficiency of production activities, thereby improving living conditions; moreover, the government is considering the application of measures to utilize ICT for the super-aging society [7]. The American Thoracic Society and European Respiratory Society recommend integrated care for patients with chronic respiratory diseases [8,9] and the application of the principle of "providing appropriate care that meets the individual needs of patients at the right time and place" [10]. Based on this principle, the practice of telemedicine has spread remarkably in Europe and the United States and has been effective in reducing home health care costs by providing the same quality of medical and nursing assistance to patients, regardless of where they reside [11]. Meanwhile, telemedicine in Japan began in the 1970s, and since then, the foundation of telemedicine has been established through tele-imaging and tele-intraoperative pathology diagnosis between distant doctors, telemedicine between distant doctors and patients [12-15], and tele-nursing, in which nurses provide health consultation to patients [16-18]. Tele-nursing, in particular, involves biometric data collection for the purpose of improving the health of patients in need of health management and provides opportunities for accurate health counseling and guidance as well as an understanding of patient health status [19]. In addition, the practice of telemedicine is gradually becoming more widespread because it can provide high quality health care by enabling interactive communication with patients via image and voice over a videophone to alleviate their concerns [20].

Chronic respiratory failure is defined as respiratory insufficiency that persists for more than 1 month [21]. The most common cause of chronic respiratory failure is chronic obstructive pulmonary disease (COPD), which accounts for 45% of patients with chronic respiratory failure [22]. According to a 2008 World Health Organization report [23], COPD is expected to become the third leading cause of death by 2030, and the number of patients with chronic respiratory failure is expected to increase in the future. The White Paper on Home Respiratory Care reported that many outpatients with COPD receive home oxygen therapy (HOT) and NPPV [24], indicating the advancement of home healthcare for patients with chronic respiratory failure. In addition, non-pharmacological therapies, such as respiratory rehabilitation and patient education on daily living activities, have become the mainstay of treatment, and a common treatment strategy has been established, regardless of the causative disease [23]. According to the American Thoracic Society and European Respiratory Society, respiratory rehabilitation is "a comprehensive medical intervention based on a thorough patient assessment, followed by exercise therapy, education, and behavior modification; it also involves the improvement of the physical and psychological status of patients with chronic respiratory disease and adherence to behaviors for long-term health promotion. NPPV is defined as the use of a nasal-mouth mask interface primarily during sleep, as a form of ventilatory support therapy for chronic respiratory failure, to reduce arterial blood gas levels, dyspnea, and morning headache and fatigue. Since it does not require tracheal intubation, patients can lead their daily lives without problems such as difficulty in speaking, eating, and drinking. The introduction of NPPV in patients with chronic respiratory failure has improved the life expectancy and significantly improved the quality of life (QOL) [25] of patients. However, chronic type II respiratory failure does not only involve hypoxemia due to impaired gas exchange, but also coexists with hypercarbia due to ventilatory failure [21]. In addition, patients with chronic respiratory failure receiving NPPV experience a decreased range of activity due to respiratory symptoms and pain when wearing masks, which is a disincentive to utilizing NPPV equipment and increases the risk of carbon dioxide (CO_2_) narcosis [27]. Patients with type II respiratory failure may be perceived as living the same daily life as patients with type I respiratory failure; however, in addition to developing hypoxia, they are prone to developing hypercarbia and therefore must learn to manage their more complex physical condition. It is assumed that patients who easily increase their oxygen level to cope with dyspnea or shortness of breath during an acute exacerbation may induce a high CO_2_ state, thereby triggering CO_2_ narcosis. Acquiring the knowledge and appropriate behavior to cope with respiratory symptoms will reduce the chance of inadvertent CO_2_ narcosis. In addition, patients with chronic respiratory failure receiving NPPV have poor respiratory function reserve and exercise tolerance and thus can easily develop acute exacerbations due to the common cold [23]. This can lead to deterioration of cardiac failure and other systemic conditions, which can be immediately fatal. Patients with chronic respiratory failure have respiratory symptoms, such as cough, sputum production, and dyspnea; hence, it is difficult to notice changes in acute exacerbations, which may lead to a delay in seeking medical attention, resulting in a more serious situation. Prolonged or repeated hospitalization leads to a decline in activities of daily living (ADLs), which in turn leads to a decline in QOL [25].

Acute exacerbation in patients with chronic respiratory failure is defined as a rapid worsening of respiratory symptoms, such as dyspnea, cough, sputum production, and respiratory failure status, and can occur when respiratory infection, air pollution, pneumothorax, right heart loading due to fatigue, psychological factors, excessive use of sleeping medication, inappropriate oxygen administration, or worsening of the underlying disease are combined to cause acute exacerbation [23] The literature detailing the causes of acute exacerbations in patients with chronic respiratory failure is scarce. According to the guidelines of COPD, a causative disease of chronic respiratory failure, 50-70% of acute exacerbations are caused by respiratory infections, including the common cold, 10% by air pollution, and approximately 30% by unknown causes [28]. Respiratory infections are caused by the indigenous bacteria *Haemophilus influenzae* and *Streptococcus pneumoniae*, whereas viral infections are caused by influenza viruses and adenoviruses. Air pollutants, such as ozone, nitrogen oxides, and suspended particulate matter less than 10 μm in diameter, can be inhaled from the air. Tobacco smoke, in particular, contains approximately 4,000 chemicals, 200 toxic substances, and more than 60 carcinogens, which become fine particles less than 2.5 μm in diameter, thereby creating an environment that can predispose patients to acute exacerbations [29]. Therefore, a smoke-free environment is required for patients with chronic respiratory failure [30]. Thus, the prevention of respiratory tract infections and avoidance of air-polluted environments are important countermeasures against acute exacerbations in patients with chronic respiratory failure. Respiratory tract infection prevention requires appropriate rest and sleep to improve the body's resistance to infection and nutritional management [23,30]. Other methods to eliminate local viruses and bacteria include wearing masks, washing hands and gargling, body hygiene, and inoculation with various vaccines [31] as specific preventive measures. Other effective measures against acute exacerbations include environmental adjustments, such as moderate temperature and humidification, the maintenance of physical activity such as respiratory rehabilitation, and drug therapy management [32-35]. In contrast, dyspnea is not a life-sustaining symptom. Nevertheless, dyspnea reportedly causes a vicious cycle of anxiety and fear about life support, leading to decreased physical activity and muscle weakness, which in turn exacerbates dyspnea [27]. Samantha et al. reported that patients with chronic dyspnea required that medical care providers furnish them with information about their daily life, such as "how to reduce shortness of breath" and "breathing exercises" [24]; thus, it can be inferred that these patients are living with anxiety while undergoing medical treatment. Samantha et al. reported that patients with chronic respiratory failure can avoid acute exacerbations through effective self-management using knowledge and skills, such as infection prevention, nutritional management, and respiratory rehabilitation, to cope with symptoms and to prolong the stable period [32].

In clinical practice, due to the shortened length of hospital stay, promotion of home care, and shortage of manpower, medical personnel provide treatment and examinations within limited time constraints; therefore, it can be inferred that information provision and health counseling with consideration for individual patients are lacking. Currently, pharmacotherapy for symptom improvement and non-drug therapies, such as information provision for knowledge acquisition and respiratory training for maintenance and improvement of physical endurance, are provided when patients are in the hospital and are continued during outpatient care. However, it has been reported that these therapies affect the course of respiratory symptoms if they are continued, not only during hospitalization but also during treatment at home [36]. Therefore, patients with chronic respiratory failure can prevent acute exacerbations and maintain their QOL if they understand and agree with the treatment at home, practice self-management, and lead their own daily lives. In Japan and overseas, there have been reports [37-39] on the implementation of respiratory rehabilitation and action plans for patients with chronic respiratory failure receiving HOT by nurses, and telemedicine services such as tele-nursing have been shown to be effective in preventing acute exacerbations and improving physical activity [16]. However, tele-nursing for patients undergoing NPPV for chronic respiratory failure, a more medically dependent condition, is considered to be a very important support for preventing acute exacerbations and maintaining QOL, albeit no firmly established evidence.

The purpose of this study is to examine the effectiveness of a tele-nursing intervention program to support the lives of patients with chronic respiratory failure receiving NPPV, incorporating non-pharmacological therapies such as proactive symptom management and infection prevention behaviors according to their physical and mental statuses. Currently, the average age of patients with chronic respiratory failure receiving NPPV is 72.6 years, and 80% of all patients are aged older than 60 years [24]. The aging of patients suggests that not only medical intervention but also social support is needed. However, there is no item in the criteria for caregiver certification that directly determines respiratory impairment; furthermore, patients with chronic respiratory failure can move if they exert themselves, and therefore, their caregiver certification tends to be lower than that of patients with other physical disabilities, thereby making it difficult to increase the amount of care support they need adequately. Given the possible decline in visual acuity and cognitive ability associated with population aging, life support tools that can more easily and clearly indicate changes in physical symptoms and are tailored to the physical condition ascertained are necessary for appropriate self-management [40]. Currently, patients with chronic respiratory failure under home care keep a medical diary to manage their physical condition, although it is necessary to apply electronic tools that provide remote patient guidance and health counseling while visualizing physical activity, vital signs, and other physical conditions to make patients more aware of the effects of treatment; moreover, educational tools that make it easier for patients to acquire knowledge and skills and to perform self-monitoring are also needed. Therefore, an electronic medical care diary should be created to enable patients to visualize their daily physical condition, and lifestyle guidance tailored to their individuality using the electronic medical care diary should be included in the tele-nursing intervention program. The information obtained from the electronic medical journal is shared between the patient and nurse, and when necessary, the patient can use the social networking service (SNS) function to communicate and to consult with the nurse via video call, in order to alleviate patient anxiety and fear during daily medical care. This is a necessary part of a tele-nursing intervention program. This study will enable patients receiving NPPV for chronic respiratory failure to safely self-manage their condition at home, thereby reducing dyspnea occurrence, improving health-related QOL, and reducing the number of readmissions and occasional outpatient visits due to acute exacerbations, as well as readmission and occasional visit rates.

# Scientific rationale of the study

Chronic respiratory failure is a condition in which respiratory failure is caused by irreversible changes in the respiratory organs, such as the lungs and airways, and persists for more than 1 month. Type I respiratory failure requires oxygen therapy, whereas type II respiratory failure requires ventilatory support therapy in addition to oxygen therapy. With the development of medical care in recent years, oxygen therapy and assisted ventilation therapy applications in home care settings have become remarkably widespread and are indispensable for maintaining and improving QOL. Patients with chronic respiratory failure have poor respiratory reserve and are prone to developing acute exacerbations; thus, respiratory rehabilitation and continued self-management after discharge from the hospital are essential for improving QOL, along with medical device management. Patients with chronic respiratory failure have dyspnea as the main symptom; however, dyspnea does not interfere with ADLs, and thus, they are able to receive home care through the management of their own health condition. The number of patients with chronic respiratory failure receiving home care is increasing, and the key to preventing acute exacerbations and maintaining patient health status is to continue self-monitoring, coping with dyspnea, and infection prevention. This study visualizes the physical condition of patients with chronic respiratory failure using a tablet terminal to help them manage their own health condition. We will develop a tele-nursing intervention program that provides remote monitoring, health counseling, and information and verify the effectiveness of the program in preventing acute exacerbations of chronic respiratory failure. For patients with chronic respiratory failure, repeated acute exacerbations cause a decline in physical activity and respiratory reserve, leading to a decline in QOL. In addition, this study is economically significant because it will help reduce medical costs by preventing acute exacerbations and re-hospitalization of patients with chronic respiratory failure via the enhancement of their home care maintenance. The ethical, scientific, and medical appropriateness of conducting this study will be reviewed by the Ethics Committee of Tohoku University Graduate School of Medicine, and approval will be obtained from the Director of the research institution.

# Selection policy for research participants

# Eligibility criteria

We will include patients with chronic respiratory failure undergoing NPPV, attending the outpatient Department of Respiratory Medicine at Tohoku University Hospital, and who have given consent to participate in the study based on the approval of the Ethics Committee of Tohoku University Graduate School of Medicine and Ethics Committee of each cooperating institution until March 31, 2019. Patients who meet all of the following eligibility criteria will be included.

(1) Patients with chronic type II respiratory failure lasting at least 1 month (with an arterial blood partial pressure of CO_2_ of 45 mmHg or higher), whose primary illness is a respiratory disease such as COPD or pulmonary tuberculosis sequelae, and who are receiving NPPV therapy [2].

(2) Since this study does not identify the type of respiratory disease, no disease stage can be established.

(3) Age at enrollment will be 20 years or older, and no upper age limit will be set, as the study will be open to anyone who is able to handle a tablet device.

(4) Gender is not an issue, as we will not alter the intervention based on gender.

# Exclusion criteria

The exclusion criteria are set as follows: patients who (1) cannot communicate due to cognitive dysfunction, (2) cannot speak Japanese, (3) cannot make outpatient visits, and (4) are aged under 20 years.

Cognitive dysfunction may render it impossible for patients to communicate with the researcher and to operate the tablet device. We considered that the inability to speak Japanese and cognitive dysfunction would affect patient evaluation because the patients would be unable to communicate with the researcher. In addition, the inability to go to the hospital as an outpatient was considered to affect the evaluation, since the patient’s ADLs had declined, resulting in an inability for the patients to perform self-care and coping behaviors on their own.

# Methods and duration of the study

# Research design

1. Research design

This is a randomized, controlled trial conducted on two groups of patients: an intervention group using an ICT-based tele-nursing intervention program for patients with chronic respiratory failure receiving NPPV at home (in addition to usual care) and a control group receiving usual care.

1. Rationale for setting up the study design

The purpose of this study is to evaluate the effectiveness of a tele-nursing intervention program for patients with chronic respiratory failure receiving NPPV. To this end, the study design was set up as a randomized, controlled trial based on the hypothesis that the group of patients who undergo the tele-nursing intervention program (intervention group) will have fewer temporary outpatient visits (percentage and number of visits) due to acute exacerbations during the intervention period than the group of patients who receive only usual care (control group).

# Overview of the distance nursing intervention program and observations

The tele-nursing intervention program for the prevention of acute exacerbations in patients with chronic respiratory failure undergoing NPPV uses a tele-nursing system developed by the researcher based on preliminary findings and literature review, in which patients with chronic respiratory failure undergoing NPPV, according to their individual physical and mental statuses, can proactively perform self-monitoring. The nursing intervention program is aimed at preventing acute exacerbations of chronic respiratory diseases by incorporating self-management strategies, such as coping behaviors for symptoms, into the lives of patients undergoing NPPV, based on their individual physical and mental conditions. This program is conducted in collaboration with the physician in charge of the patient, as it includes information that can be utilized for the patient’s medical treatment, although it is an action outside of normal medical treatment. The program mainly consists of (1) a remote monitoring of the participant's daily physical condition, (2) health counseling on questions and problems in the participant's daily life, and (3) the provision of information on lifestyle and self-management to reduce the occurrence of respiratory symptoms. Moreover, the program has the following components: (1) data input using tablet terminals from the homes of patients with chronic respiratory failure receiving NPPV, (2) remote monitoring and health counseling from the researchers' PC terminals, (3) a database server, (4) the attending physician of the Department of Respiratory Medicine at Tohoku University Hospital, and (5) cell phone communication. Tablet terminals will be loaned by the researcher. Once a day, at a particular time, the participant uses approximately 10 min to respond to questions displayed on the tablet terminal from a list of options, and data on his/her physical condition are sent to the server. The data include vital signs, respiratory symptoms, food intake status, defecation status, medication status, physical symptoms other than respiratory symptoms, and the patient’s questions to the medical staff. After the patient enters vital signs and other measurement information into the tablet, the transmitted data are immediately monitored, and triage is performed based on the trigger points for each question item and nursing responses that have been set in advance with the physician in charge. If triggers are applicable, direct physical and mental assessment is performed by telephone or videophone according to the nursing responses that have been set, and nursing support is provided based on the Pulmonary Rehabilitation Manual [30]. If no triggers are applicable, the process is terminated by message delivery. There are 24 trigger items in total. One of the trigger items is transcutaneous oxygen saturation (SpO_2_); if the SpO_2_ is above 95%, the trigger is not applicable and no nursing support is provided. If the SpO_2_ is between 94% and 90%, the trigger is set as "alert" and the patient's condition is confirmed together with other data. If the SpO_2_ is 89% or lower, the trigger is set as "applicable"; the patient's condition is confirmed, and nursing care is provided using a videophone. The response threshold and nursing response corresponding to each question item are set individually based on the participant’s condition. As an example of the nursing response, the cause of the SpO_2_ decline is assessed based on the patient's physical and mental condition, and health counseling and information are provided via a videophone on the tablet terminal screen to enable appropriate self-management actions such as medication, NPPV application, response to shortness of breath (breathing techniques), and infection prevention actions in accordance with the cause of SpO_2_ decline. After the nursing response, the details of the response are reported to the physician in charge by phone or e-mail, and the response for the following day and beyond is discussed. If the patient is unsure about the situation after receiving nursing care via videophone, the physician in charge is promptly contacted via phone call to discuss how to handle the situation. Videophone communication with the participant is conducted at the participant’s discretion or when triggered, based on the data entered on the tablet device. The tablet terminal input time period is from 8:00 am to 12:00 pm, and the nursing response is expected to take place from 1:00 pm to 5:00 pm, once a day for approximately 30 min. If the tablet device is not input for more than 2 days, a phone call will be immediately made to the target participant to resolve the cause of the problem. The server will be set up by a company that produces this system, and Secure Sockets Layer encryption will be used for transmitted data, with access to the server limited to researchers and physicians at cooperating medical institutions. The communication technology will be the Long Term Evolution service, a cell phone communication network, under a general contract with NTT DoCoMo. We considered this service to be optimal for this study because of its robust information leakage countermeasures using proprietary security features. The tablet terminals will be loaned by the researcher and will be returned after the intervention is completed.

The web-based program in the tele-nursing system consists of a patient site and a researcher site. The tablet terminal at the patient site has (1) a screen for recording daily physical condition, (2) a screen for video calls, (3) a screen for SNS input, and (4) a screen for respiratory rehabilitation information. The researcher's site will have (1) a list of the participant's telemonitoring data and (2) a screen for inputting comments. The researcher can check the participant's daily records and output the changes over time on a summary sheet.

The intervention period will be 24 weeks, and the number of ad hoc visits and readmissions during the past 12 and 24 weeks (primary endpoints) will be examined by the researcher at enrollment, 12 weeks after enrollment, and 24 weeks after enrollment, using the electronic medical record. At enrollment and at regular outpatient visits 12 and 24 weeks after enrollment, researchers will conduct a questionnaire survey about daily life, and perform respiratory function and 6-minute walk tests. In addition, activity meters will be checked 12 and 24 weeks after enrollment to collect data on the average number of steps and patient activity. Respiratory function and 6-min walk tests at enrollment as well as 12 and 24 weeks after enrollment will be performed by the researcher using equipment purchased using the research fund, since these tests are not covered by insurance and are outside the scope of usual medical care. Since the primary endpoints of this study are the number of emergency visits and rehospitalizations due to acute exacerbations of chronic respiratory diseases, the intervention will not be stopped in the event of worsening symptoms due to acute exacerbations. If the participant dies, the intervention is terminated because the study cannot continue. If a participant requests that the intervention be continued after their participation in the program, the intervention may be offered for an extended period of time, regardless of whether the participant is in the intervention or control group.

# Combined therapy

1. Allowable concomitant therapy

The interventions in this study will mainly consist of telemonitoring, information provision, and health counseling. The program will not interfere with by other therapies, as it is important for patients to successfully continue their existing therapies in the home care setting. Therefore, any other concomitant therapies that the patient is taking outside of the program will be allowed.

1. Unacceptable concomitant therapy

There are no specific concomitant therapies that are not acceptable.

# Survey Schedule

|  | **Evaluation item** | **Survey schedule** | | |
| --- | --- | --- | --- | --- |
|  |  | **At enrollment**  **(Baseline)** | **12 weeks after enrollment** | **24 weeks after enrollment** |
| **Interview or medical record** | Basic patient information | ◯ |  |  |
|  | Occasional visits and readmissions in the past 12 weeks | ◯ | ◯ |  |
|  | Occasional doctor visits and readmissions in the past 24 weeks | ◯ |  | ○ |
| **Questionnaire** | SGRQ | ◯ | ◯ | ◯ |
|  | EQ-5D | ◯ | ◯ | ◯ |
|  | SCAQ | ◯ | ◯ | ◯ |
| **Objective assessment items** | Respiratory function test | ◯ | ◯ | ◯ |
|  | 6-min walk test | ◯ | ◯ | ◯ |
|  | Average number of steps, activity level |  | ◯ | ◯ |

# Research period

The research period will be from May 2017 (after approval by the Ethics Committee of Tohoku University Graduate School of Medicine) to March 2019.

# Adverse event assessment

# Obtaining information

1. If a serious adverse event/failure occurs, the investigator will take appropriate action and report it to the principal investigator at the research institution.
2. The principal investigator of the research institution confirms the following items to the research participant.

**Items to be Confirmed by the Principal Investigator to the Researcher.**

| 1) Name of adverse event/failure  2) Severity classification [1]  3) Severity [2], reason for judging as serious  4) Predictability (unknown/known) [3]  5) Causal relationship with the intervention (study drug/device)  6) History of the event/defect (date of onset, course, outcome, and others)  7) Information regarding the participant's identification (initials, age, and gender) |
| --- |

**Severity Classification [1].**

I judge according to the National Cancer Institute Common Terminology Criteria for Adverse Events (NCI CTCAE v4.0: <http://www.jcog.jp/doctor/tool/CTCAEv4J_20150310.pdf>). If there is no item that corresponds to the NCI CTCAE Classification, the judgment should be made with reference to the following: "Criteria for Classification of Severity of Adverse Events."

| **Severity classification (NCI CTCAE grade）** | **Criteria** |
| --- | --- |
| **Mild (Grade 1)** | No symptoms or mild symptoms. Clinical or laboratory findings only.  No treatment required. |
| **Moderate (Grade 2)** | Requires minimal/local/noninvasive treatment.  Age-appropriate limitation of activities of daily living (ADLs) other than personal care*. |
| **Severe (Grade 3)** | Severe or medically significant, but not immediately life-threatening.  Requires hospitalization or prolonged hospitalization. Inability to perform activities/behaviors. Limitation of ADLs**. |
| **Most severe (Grade 4)** | Life-threatening. Requires immediate medical attention. |
| **Death (Grade 5)** | Death due to adverse event. |

*Instrumental ADLs (ADLs other than personal care): Meal preparation, shopping for daily necessities and clothing, using the telephone, managing money, and others.

**Self-care ADLs: The patient is able to bathe, to dress/undress, eat, to use the toilet, and to take medications; patient is not bedridden.

**Definition of Serious Adverse Events [2].**

| 1) Causing death  2) Life-threatening  3) Requiring hospitalization or prolonged hospitalization for treatment  4) Permanent or significant disability or dysfunction  5) Resulting in congenital anomalies in offspring. |
| --- |

Hospitalization stipulated in the research protocol, hospitalization to perform a therapy or examination planned before the research (before obtaining consent) and during the research (scheduled surgery, examination, and others), and hospitalization for therapy or examination not associated with the adverse event (medical examination and others) will not be considered as serious adverse events.

**Definition of Predictability (Unknown/Known) [3].**

| **○Unpredictable (unknown)**  The occurrence of the event or trend of occurrence, such as the number and frequency of occurrences, as well as conditions of occurrence, cannot be predicted from the official documents (package inserts, papers, and others) related to the study drug/device in question.  **○Predictable (known)**  The occurrence of the event or trend of the number and frequency of occurrences, as well as conditions for occurrence, can be predicted from the official documents (same as above) related to the study drug/device in question. |
| --- |

# Description of adverse events

Since this study will be an informational and health counseling activity for patients, adverse events from the intervention are likely to be very minimal. However, since respiratory function and 6-min walk tests will be performed during the measurement of secondary endpoints, it is possible that minor adverse events such as temporary worsening of symptoms may occur during these tests. In the event of an adverse event, the attending physician should be notified immediately for necessary action. For all adverse events, the progress during the intervention should be described in a case report, including the name of the adverse event, its severity, the reason it was deemed serious, the date of onset, the date of outcome, the treatment, the outcome, and the causal relationship with the intervention.

# Response to serious adverse events/failures (including the scope of adverse events to be reported to the head of the research institution)

# Response to adverse events/failures

1. If an adverse event/failure occurs, the investigator will take appropriate measures to investigate the cause, paying attention to ensuring the safety of the research participants [2]. This study will include a 6-min walk test as an endpoint. Since the 6-min walk test in this study is an outpatient examination, we will set up a pre-established system with the physician in charge and outpatient nurses to enhance patient safety and to promptly provide appropriate medical care in the event of an exacerbation of respiratory failure. In addition, a system will be established such that the same response can be provided to the physicians in charge at the collaborating research facilities, and efforts will be made to ensure participant safety.
2. As much as possible, the investigators will continue to follow up and to confirm the outcome of any symptoms or abnormal fluctuations in laboratory test values until, in principle, the event disappears, the patient recovers to the pre-research state, or it is determined that the event is no longer a clinical problem.
3. If the investigators determine that follow-up is unnecessary, such as when an irreversible adverse event/failure develops, follow-up will be terminated at the end of the study for the research participant; moreover, the reason for determining that follow-up is unnecessary should be stated in the comments section of the case report form.

# Report to the head of the research institution and the principal investigator

1. The principal investigator of the research institution shall report the occurrence of a serious adverse event/failure to the head of the research institution within time limits from the time when he/she becomes aware of the occurrence, as shown in the following table. The report should be made using "(Form No. 9) Report on Serious Adverse Events", which is

downloaded for clinical research from: http://www.med.tohoku.ac.jp/public/rinri_d.html.

1. In the case of a multicenter collaborative study, the principal investigator of the research institution should report the occurrence of a serious adverse event/failure to the principal investigator (principal investigator) within time limits from the time when he/she becomes aware of the occurrence, as shown in the following table. The report shall be made using the "(Reference Form 1) Report on Serious Adverse Events."
2. If the principal investigator is in another institution, the response will follow the procedures of the concerned institution.

**Criteria and Deadlines for Reporting to the Head of the Research Institution and Principal Investigator**

|  | **Mild/Moderate/Severe (Grade 1/2/3)** | | | **Most severe (Grade 4)** | | **Death** | | **Other medically important conditions** |
| --- | --- | --- | --- | --- | --- | --- | --- | --- |
|  | **Predictable (known)** | **Unpredictable (unknown)** | | **Predictable (known)** | **Unpredictable (unknown)** | **Predictable (known)** | **Unpredictable (unknown)** |  |
|  | **Hospitalization (No / Yes)** | **Hospitalization (No)** | **Hospitalization (Yes)** |  |  |  |  |  |
| **Causal relationship exists** | Report not required | Report not required | First report: Within 10 days  Additional report: As and when necessary | Primary report: Within 72 days  Second report: within 7 days  Additional reports: As and when necessary | | | |  |
| **No causal relationship** | Report not required | Report not required | First report: Within 10 days*  Additional reports: As and when necessary* | Primary report: Within 72 days*  Second report: within 7 days*  Additional reports: As and when necessary* | | | |  |

*On treatment or within 30 days of last protocol treatment date only

# Reporting to collaborating institutions

1. In the case of multicenter collaborative research, the principal investigator shall report the adverse event/failure to the principal investigators of the research institution where the adverse event/failure occurred and collaborating research institutions, using the Serious Adverse Event Report Form; in addition, a report shall be sent to the head of the research institution and Ethics Committee.
2. The principal investigator of the research institution will take necessary measures in response to instructions from the head of the research institution.

# Evaluation items and methods

# Primary endpoints

Since the purpose of this study is to verify the effectiveness of a tele-nursing intervention program for the prevention of acute exacerbations of chronic respiratory diseases, it is necessary to set the status of acute exacerbations as an evaluation item. Acute exacerbations cannot be determined simply by considering the number of temporary visits and rehospitalizations separately, and thus, the primary endpoints of this study are the statuses of temporary outpatient visits (rate and number of visits) and rehospitalizations (rate and number of hospitalizations) due to acute exacerbations in the past 12 and 24 weeks, as well as the reasons for the outpatient visits or hospitalizations at enrollment (baseline) and 12 weeks after enrollment. The data will be collected from the patients themselves at baseline, 12 weeks after enrollment, and 24 weeks after enrollment. However, if the patient's complaint is unclear, it will be collected from the patient's medical record.

# Secondary endpoints

Secondary endpoints included the St. George's Respiratory Questionnaire (SGRQ) score, developed as a specific health-related QOL measure for respiratory diseases; Euro Qol 5 Dimension (EQ-5D) index, a QOL assessment instrument used to calculate quality-adjusted life years in the economic evaluation of medical technology; and Self-Care Capacity Questionnaire score, a measure of the degree of patient self-care. respiratory function measurements; and 6-min walk test distance will be collected at baseline and 12 and 24 weeks after enrollment. In addition, as an objective measure of physical activity, the average number of steps taken and amount of activity will be collected from an activity meter at 12 and 24 weeks after enrollment.

# Centralized evaluation system

This study does not use a centralized evaluation system.

# Registration and allocation

# Registration

1. Registration procedure

The attending physicians at the Department of Respiratory Medicine of Tohoku University Hospital and each collaborating institution will select the participants and distribute the survey instructions during regular outpatient visits. Participants who read the study description and are interested in the study will, of their own free will, inquire about an initial interview with the investigators. At the initial interview, the eligibility criteria will be confirmed and the survey will be explained orally and in writing; further, the participants will be enrolled in the study upon submission of a participant consent form.

1. Notes on registration

To avoid duplication of enrollees, a list of enrollees will be created and maintained. In addition, since it is anticipated that the required number of participants will not be gathered after the study begins, participants will be added to the list via a sequential enrollment method.

# Allocation

1. Assignment method and assignment adjustment factors

A table will be created in advance to randomly assign the expected number of participants to the intervention and control groups. Consenting participants will be assigned a serial number and anonymized in a consolidated manner. Research assistants will assign participants to the intervention and control groups by fitting the participants to a random number table generated from the serial numbers. Concerning allocation adjustment, since the study is a multicenter study, it will be necessary to allocate the participants to each facility, since different facilities are considered to have different medical systems.

# Number of planned cases and basis for setting

# Planned number of cases

The intervention and control groups are expected to have 17 patients each; hence, the total expected number of patients is 34.

# Basis for setting

The primary hypothesis of this study is: A group of patients with chronic respiratory failure receiving NPPV with a tele-nursing intervention program (intervention group) will have fewer temporary outpatient visits (percentage and number of visits) due to acute exacerbations during the intervention period than a control group receiving only usual care (Figure 1). Based on the literature [24], we assumed that the average number of visits to the emergency department due to acute exacerbations of chronic respiratory diseases would be 2.4 visits/year for NPPV users and 1.6 visits/year for NPPV non-users to reduce the number of outpatient visits by 1.0 visit/year. The significance level, power of the study, and standard deviation of the number of improvement of visits are 5%, 80%, and 1.0 visit/year, respectively. In addition, assuming a dropout rate of 10% of the total number of patients, the intervention and control groups were calculated to require 17 patients each, for a total of 34 expected enrollees in both groups.

# Statistical analyses

# Statistical analysis methods

Data on basic participant characteristics will be examined for normality using the Kolmogorov-Smirnov test, and the differences between the means or medians for the data in the intervention and control groups will be compared using the *t*-test, based on the normality of the data. Fisher's exact test will be used to compare the primary endpoints of temporary outpatient visit and readmission rates between groups, and student *t*-test will be used to compare the number of temporary visits and readmissions between groups. Paired *t*-tests will be used to compare changes in secondary endpoints at baseline, 12 weeks after enrollment and intervention initiation, and 24 weeks after enrollment and intervention initiation (at the end of the intervention period). The analysis will be conducted using a personal computer with Microsoft Windows 8.1 OS installed and IBM SPSS version 21.0 statistical software, with a two-tailed significance level of 5%.

# Interim analysis and early termination of studies

An interim analysis will be conducted 12 weeks after the start of the intervention and enrollment to determine if the main objective has been achieved. The same methods of analysis will be used as for the main analysis. Even if the planned number of study participants is not attained as a result of the interim analysis, the study may be terminated early, if the superiority of the intervention group is deemed sufficient. The study will also be terminated if adverse events related to the intervention occur in 10% or more of the total number of participants.

# Methods of data management and self-inspection

# CRF preparation

The researcher will complete and correct the entries on the CRF. The investigator will complete the CRF promptly after the completion of the intervention for each participant. Since the program lasts for 24 weeks, the CRF will be prepared after 24 weeks of intervention. Care should be taken to ensure that the primary and secondary endpoints are covered and that vital signs and information on how the daily evolution of the participant's condition can be clearly and concisely ascertained. In addition, as with the nursing record, videophone information should be recorded such that it can be understood.

# CRF self-inspection

1. The principal investigator shall check the contents of the CRF and the source documents (medical records, raw data, and others) for consistency.
2. If there is a discrepancy between the CRF and source documents, a report explaining the reason for the discrepancy should be prepared.
3. The principal investigator or research assistant at the research institution should inspect and confirm the contents of the prepared CRF and then affix his/her name and seal or sign it.
4. The research assistant will keep the CRF away from third parties.

# Sending and storage of CRFs

The principal investigator of the research institution shall submit the original or a copy of the prepared CRF to the data center using the established procedure "Guide to Completing the CRF"; moreover, the principal investigator shall keep a copy of the CRF. The CRF shall be submitted to the following address:

Tohoku University Hospital Clinical Trial Data Center

Address: 1-1 Seiryo-cho, Aoba-ku, Sendai-shi, Miyagi 980-8574

Tel: 022-717-7122

The data center will not send CRFs by fax, but will print out the forms and submit them in paper form or register the CRFs by electronic submission. In the case of paper CRFs, a copy of the completed CRF or an electronic copy of the scanned paper CRF should be kept by the principal investigator at the research institution.

# CRF correction procedure

When amending the CRF, the principal investigator of the research institution should prepare and submit a record of the change or amendment made on the CRF using the established procedure "Guide to Completing the CRF"; the principal investigator should keep a copy of the record.

# Procedures for obtaining informed consent

# Explanation to research participants

Before enrollment, the investigator will provide the research participant with an explanatory document approved by the research institution, explaining the following items. (Items that do not apply to this study will be clearly indicated as not applicable in the explanatory document.)

**Items to be included in the explanatory document**

(1) The title of the research and the fact that permission to conduct the research has been obtained from the head of the research institution.

(2) Research institution and principal investigator.

(3) Purpose and significance of the research.

(4) Method and duration of the research.

(5) Reason for selection as a research participant.

(6) Burden and anticipated risks and benefits to the research participants.

(7) A statement that consent to conduct or continue the research may be withdrawn at any time.

(8) A statement that research participants will not be treated disadvantageously due to non-acceptance or withdrawal of consent to conduct or continue the research.

(9) Methods of disclosing information regarding the research.

(10) Methods for obtaining and accessing research protocols and materials related to research methods at the request of research, participants within limits that do not hinder the protection of the personal information of other research participants or the securing of the originality of the research.

(11) Handling of personal information (including the method of anonymization, if any).

(12) Methods for storing and disposing of samples and information.

(13) Conflicts of interest statement (sources of funding for research, possible conflicts of interest, and involvement of researchers and other related organizations).

(14) Responses to consultations with research participants and other relevant parties.

(15) Details of any financial burdens or gratuities to research participants.

(16) In the case of invasive research, whether or not compensation will be provided for damage to health caused by the research, and if so, the nature of such compensation.

(17) If there is a possibility that samples or information obtained from research participants will be used for future research that is not specified at the time consent is obtained from the research participants or will be provided to other research institutions, the details of the possibility of such use or provision should be mentioned at the time consent is obtained.

# Agreement

After explaining the study, the participant is given sufficient time to think about it; it is confirmed whether the participant understands the study before being asked to participate in the study. If the research participant agrees to participate in the study, the consent document will be signed by the research participant. A copy of the consent document is kept by the principal investigator at the research institution, and another copy is given to the research participant.

# Procedures for obtaining consent by proxy

If the participant is not cognitively impaired and has difficulty in writing due to physical disability, consent shall be obtained by a substitute scribe. The substitute scribe should be a person who lives with the participant and is able to communicate well with the participant. The explanation and requirements for obtaining consent by a substitute scribe shall be the same as those for the participant. When a substitute scribe writes on behalf of the participant, the participant's consent should be recorded separately.

# Handling of personal information

# Purpose of use of personal information

To obtain adequate study results, personal information will be used to identify and to examine individual research participants not only during treatment but also for a long period of time after the end of treatment, and to properly manage the information obtained.

# Method of use of personal information (method of anonymization)

The research participants' personal information will be protected by linkable anonymization, and the list of linkable anonymizers will be kept in a locked cabinet together with the USB device. I will use my research participant IDs, and no other personal information will be disclosed from the research institution to the data center. Medical record numbers will be converted to research participant IDs and managed using a correspondence table.

# Safety management responsibility system (safety management measures for personal information)

The principal investigator of the research institution will take safety control measures in handling personal information to minimize the risk of information leakage. To ensure that participant data is sent and received securely over the cell phone network, a server will be installed at a system development company that has been granted the Privacy Mark certification for data acquisition, storage, and disposal. The ICT used by NTT DoCoMo Tohoku in this program has already been used to remotely monitor the lives of older local residents and its own security system has been established; therefore, the risk of information leakage was considered low; no information provision to NTT DoCoMo was assumed. During data communication, a virtual private network connection is used, and the system is set up in such a way that data leakage or falsification by a third party is impossible. The intervention is conducted in a room where privacy is maintained (such as a graduate student's room in the Department of Cancer Nursing, Tohoku University Graduate School of Medicine), and access to the room is restricted to researchers only during data viewing, telephone calls, and videoconferencing. The details of the intervention will be promptly reported to the attending physician, who will be authorized to view the participant data, and the intervention will be conducted in collaboration with the attending physician. The obtained participant data will be handled in the graduate student room. When saving the data on the USB device, a security lock should be implemented, and the USB device should be stored in a locked cabinet.

# Burden on research participants, anticipated risks (including possible adverse events) and benefits, a comprehensive assessment of these risks, and measures to minimize the burden and risks

# Summary of anticipated benefits and disadvantages associated with participation in the study

(1) Predicted benefits

The tele-nursing intervention program used in this study is a nursing support method that may be applicable to the participants and is expected to improve their health status after the intervention. In addition, although the evaluations (respiratory function test and 6-min walk test) are performed outside of routine medical care, researchers will prepare the equipment and perform the tests, such that there will be no financial burden on the participants. In addition, there are no special medical practice or economic benefits to be gained by the participation of the research participants in this study compared with routine medical care.

(2) Anticipated risks and disadvantages

The tele-nursing intervention program used in this study is a non-invasive intervention and thus is expected to have few intervention-related adverse events. The anticipated disadvantage of participating in this study is a delay in receiving medical attention when a patient's condition changes. Since this program is designed for patients whose general condition is stable, the tablet terminal is not equipped with a notification function in the event of a sudden change in participant condition. Therefore, in the event of a sudden change in condition, the participant must request an ambulance immediately or visit a hospital on his/her own. The participant and his/her family will be fully informed of this, and their consent will be obtained after understanding the situation. If a change in the participant's condition is evident, the attending physician or the physician in charge should be consulted immediately for appropriate measures.

# Methods of storage and disposal of samples and information

# Storage of samples and information

The principal investigator shall store the samples and information as shown in the table below.

| **Preserved by** | **Samples and information to be stored** | **Retention period** |
| --- | --- | --- |
| **Principal investigator** | ○Research-related documents or source documents to be preserved at the research institution   1. Basic information on patient background 2. Daily vital sign data 3. Respiratory function and 6-minute walk test measurements   ○Procedure manual | 5 years after completion of the study |

# Disposal of samples and information

When disposing of samples and information obtained from human participants, the principal investigator shall anonymize the participants.

# Sources of funding for the research and research institution and researcher conflicts of interest

This research is funded by the Ministry of Education, Culture, Sports, Science and Technology of Japan, a grant-in-aid for scientific research for the "Development of a New Remote Nursing Support Program Using Visualization of Physical Activity for Patients with Chronic Respiratory Failure" (Research Project No. 16K20757), and a research investigation grant from the Telecommunications Dissemination Foundation. There is no relationship with any company in this research that would amount to a conflict of interest.

# Intellectual property

The results, data, and intellectual property rights obtained from this research will belong to Tohoku University and the principal investigator. Specific data handling and allocation will be decided upon consultation. Whether the intellectual property of the principal investigator belongs to the individual or to the research institution is subject to an agreement between the principal investigator and research institution with which he/she is affiliated.

# Methods of disclosing research-related information

# Registration of the research plan

The principal investigator will register the research outline on the website of the University Hospital Medical Information Network (<http://www.umin.ac.jp/ctr/index-j.htm>), a public database, and update it as appropriate based on changes in the research protocol and research progress.

# Registration of research results

The principal investigator will register the research results in a public database after the research is completed. However, matters pertaining to the human rights of research participants, human rights of researchers and other related parties, and protection of intellectual property as well as matters that are permitted by the head of the research institution based on the opinion of the Ethics Committee shall not be disclosed, as these matters would significantly impede the research from the perspective of personal information protection.

# Publication of research results

Upon completion of the research, the principal investigator will promptly publish the research results in a medical journal or similar publication platform, after taking measures to protect the personal information of the research participants. The head of the research institution will be promptly informed when the study is published.

# Report content and method of reporting to the head of the research institution

The principal investigator reports the following information to the head of the research organization by means of "Report on Research Progress” (Form No. 8).

1. Progress of the research
2. Status of occurrence of research-related adverse events
3. Summary of study results

# Responding to consultations from research participants and their concerned parties

Contact for general research inquiries and privacy policy inquiries

Research Secretariat

Doctoral Program: Makoto Shimoyama

Tohoku University Graduate School of Medicine, Department of Health Sciences, Family Support Nursing, Cancer Nursing

Tel: 090−7798−1240

E-mail:　[makoto.shimoyama.r7@dc.tohoku.ac.jp](mailto:makoto.shimoyama.r7@dc.tohoku.ac.jp)

# A detailed statement on any financial burden or honorarium to the research participants

The anticipated economic burden on the research participants will be the cost of recharging tablet batteries, which will be incurred because of the use of tablet terminals. Assuming a battery capacity of 1000 mAh, a 100% charging efficiency, and an electricity rate of 24 yen per kWh, the cost per charge would be approximately 0.88 yen. If the batteries are recharged approximately once daily, the total recharge cost would be 0.88 yen $\times$180 days or approximately 160 yen. This out-of-pocket expense will be fully explained to the participants, and their consent will be obtained. We do not anticipate any financial rewards to participants after the research.

# A statement on whether or not compensation is provided for research-related health hazards and the nature of such compensation for research involving invasive procedures

If a research participant suffers health problems as a result of the conduct of the research, the research institution will provide medical treatment and take other necessary measures. If the health condition of a research participant deteriorates, the research institution will promptly recommend that the research participant seek medical attention and will ensure that the research participant receives appropriate treatment from the attending physician or physician in charge. The research participant will be responsible for paying his/her own share of medical expenses.

# A statement describing the possibility that the sample/information may be used for future research or provided to other research institutions

We do not anticipate the use of data obtained in this study for secondary purposes other than this study. No ancillary research is planned at this time; nevertheless, if ancillary research is planned in the future, a new research protocol will be prepared and the research will be conducted after a new ethics committee approval.

# Provision of samples and information to collaborating research institutions of this research or other research institutions

Samples and information obtained in this research will not be provided to other research institutions.

# Provision of samples and information to commercial entities

Samples and information obtained in this research will not be provided to other commercial entities.

# Changes to the research protocol

When amending the research protocol, the principal investigator shall obtain approval from the head of the research institution after review by the Ethics Committee.

Changes to the content of the research protocol are handled under two categories: amendments and revisions. Other additions of supplementary explanations that do not fall under the abovementioned categories of changes will be distinguished as memorandum.

(1) Amendment

Amendments to the research protocol that may increase the risk to research participants or affect the primary endpoints and require institutional approval. The following cases are applicable:

1) Changes that increase the burden on participants (increase in the invasiveness of procedures, such as blood sampling and tests)

2) Changes in exclusion criteria due to the occurrence of serious adverse reactions

3) Changes in the evaluation method of efficacy and safety

4) Changes in the number of cases

(2) Revisions

A change in the research protocol that is not likely to increase the risk to research participants and does not affect the primary endpoint. Revisions require approval by the respective research institutions. The following cases are applicable:

1) Changes that do not increase the burden on participants (changes in the timing of testing)

2) Changes in the research period

3) Change of the researcher

(3) Memorandum

Memorandums are explanations of the research protocol distributed by the principal investigator to research personnel, not to change the content of the research protocol, but to reduce variations in the interpretation of the text and to call for special attention.

# Research implementation structure

# Name of the research institution and principal investigator

Research Organization: Division of Cancer Nursing, Department of Family Support Nursing, Tohoku University Graduate School of Medicine

Research Director: Professor Fumiko Sato

Tel: 022-717-7926; Fax: 022-717-7910

E-mail:　fsato@med.tohoku.ac.jp

# Joint research institutions

1. Joint research institutions: Yes

○ If the name of the institution can be identified:

Collaborating institutions with which future participation is anticipated: Tohoku University Hospital and Department of Respiratory Medicine, General Hospital to which the participant is referred from the Department of Respiratory Medicine, Tohoku University Hospital.

| **Name of Institution** | **Researcher** | **Availability of the Ethics Committee** |
| --- | --- | --- |
| Tohoku University Hospital | Dr. Hiromasa Ogawa, Respiratory Medicine | Yes |
| Tohoku Medical and Pharmaceutical University Wakabayashi Hospital | Dr. Tsuneyuki Takahashi, Respiratory Medicine | Yes |
| Ishinomaki Red Cross Hospital | Dr. Seiichi Kobayashi, Respiratory Medicine | Yes |
| Tohoku Rosai Hospital | Dr. Yusuke Tashiro, Respiratory Medicine | Yes |
| Sendai Medical Center | Dr. Yu Miki, Respiratory Medicine | Yes |
| South Miyagi Medical Center | Dr. Shinji Okada, Respiratory Medicine | Yes |
| Osaki Citizen Hospital | Dr. Ryutaro Igusa, Respiratory Medicine | Yes |

○ If the research institution in question has its own Ethics Committee:

After approval by the Ethics Committee of Tohoku University Graduate School of Medicine, the physician who will be the principal investigator will be promptly notified, and approval will be obtained from the Ethics Committee of each cooperating institution.

○ If there is no Ethics Committee at the research institution:

The research institution requests the Ethics Committee of Tohoku University Graduate School of Medicine to review the protocol and to provide approval.

(2) Role of collaborating institutions: Referral of participants, handling of adverse events

(3) Anonymization upon collection of samples and information

(Reason: Anonymization will be performed at the participant’s institution to protect the participant’s personal information.)

(4) When anonymization is performed

Anonymization organization: The collaborating institution where the sample/information was collected and our institution (Reason: To protect patients' personal information at the participant institution)

(Reason: To protect patients' personal information at the patient’s institution.)

(5) Confirmation of the contents of informed consent at other institutions

(Reason: All informed consents at other institutions will be given by the research collaborator; hence, the contents of such consents have already been confirmed.)

(6) Samples/information will not be provided to the collaborating institution.

# Role of the research secretariat and researchers

(1) Research Bureau, Experimental Machine Manager

Postdoctoral Program: Shin Frost Mountain

Tohoku University Graduate School of Medicine, Department of Health Sciences, Family Support Nursing Course

Tel: 090-7798-1240 ac

E-mail: [makoto.shimoyama.r7@dc.tohoku..jp](mailto:makoto.shimoyama.r7@dc.tohoku.ac.jp)

(2) Research coordinators

Postdoctoral Program: Shin Frost Mountain

Tohoku University Graduate School of Medicine, Department of Health Sciences, Family Support Nursing Course

Tel: 090-7798-1240

E-mail: makoto.shimoyama.r7@dc.tohoku.ac.jp

Associate Professor Hiromasa Ogawa

Department of Respiratory Medicine, Tohoku University Hospital/Division of Industrial Medicine, Graduate School of Medicine, Tohoku University

Tel: 022-717-7874

E-mail: ogawa-hiro@m.tohoku.ac.jp

Assistant Professor Teruyuki Sato

Tohoku University Graduate School of Medicine, Department of Internal Medicine, Department of Pathology, Division of Respiratory Medicine

Tel: 022-717-8539

E-mail: [satoteruyuki@gmail.com](mailto:satoteruyuki@gmail.com)

(4) Person responsible for storage and management of samples and information

Professor Fumiko Sato

Department of Cancer Nursing, Division of Family Health Nursing, Tohoku University Graduate School of Medicine

Tel: 022-717-7926; Fax: 022-717-7910

E-mail: [fsato@med.tohoku.ac.jp](mailto:fsato@med.tohoku.ac.jp)

# Statistical analysis and data center

(1) Person responsible for statistical analysis and data management

Professor Tomiko Sato

Department of Cancer Nursing, Division of Family Support Nursing, Department of Health Sciences, Tohoku University Graduate School of Medicine

Tel: 022-717-7926; Fax: 022-717-7910

E-mail: fsato@med.tohoku.ac.jp

(2) Data management staff:

Doctoral Program: Makoto Shimoyama

Tohoku University Graduate School of Medicine, Department of Health Sciences, Family Nursing, Cancer Nursing

Tel: 090-7798-1240

E-mail: [makoto.shimoyama.r7@dc.tohoku.ac.jp](mailto:makoto.shimoyama.r7@dc.tohoku.ac.jp)

# Contact for research inquiries

1. How to enroll research participants and how to respond to adverse events

Doctoral Program: Makoto Shimoyama

Tohoku University Graduate School of Medicine, Department of Health Sciences, Family Support Nursing, Cancer Nursing

Tel: 090−7798−1240

E-mail:　[makoto.shimoyama.r7@dc.tohoku.ac.jp](mailto:makoto.shimoyama.r7@dc.tohoku.ac.jp)

# References

1. Ministry of Health, Labour and Welfare. White Paper on Health, Labour and Welfare in 2016-A Social Model to Overcome Population Aging, viewed December 5, 2016; 2016. Available from: http://www.mhlw.go.jp/wp/hakusyo/kousei/16/dl/all.pdf.

2. Ministry of Education, Culture, Sports, Science and Technology. Lifelong Learning in a Longevity Society – Happiness to Learn for 100 Years of Life at Any Age "Happy Aging Society" - viewed; 2012. [Cited December 5, 2016]. Available from: http://www.mext.go.jp/component/a_menu/
education/detail/__icsFiles/afieldfile/2012/03/28/1319112_1.pdf.

3. Study Group on Appropriate Medical Care Provision for the Elderly. Guidelines for appropriate medical care provision for the elderly. J Jpn Geriatr Soc. 2014;51: 89-96.

4. Headquarters for the Promotion of Social Security System Reform; 2015, First Report of the Expert Committee on the Promotion of Reform through the Utilization of Medical and Nursing Care Information: In estimating the number of hospital beds by medical function and formulating a regional medical care plan, viewed. jp/jp/singi/shakaihoshoukaikaku/houkokusyo1.pdf. [Cited December 5, 2016]. Available from: http://www.kantei.go.

5. National Council for Social Security System Reform, Report of the National Council for Social Security System Reform – A path to pass on reliable social security to future generations -, viewed. [Cited December 5, 2016]. Available from: http://www.kantei.go.jp/jp/singi/kokuminkaigi/
pdf/houkokusyo.pdf; 2013.

6. Ministry of Health, Labour and Welfare. Promotion of ICT in the Health, Medical and Nursing Care Fields, viewed December 5, 2016; 2014. Available from: http://www.mhlw.go.jp/file/06-Seisakujouhou-12600000-Seisakutoukatsukan/0000042495.

pdf.

7. Ministry of Internal Affairs and Communications. [Cited December 5, 2016]. Available from: http://www.soumu.go.jp/main_content/000268318. pdf, Realization of "Smart Platinum Society" - viewed; 2013, ICT Super-Aging Society Initiative Council Report.

8. Nici L, ZuWallack R, American Thoracic Society Subcommittee on Integrated Care of the COPD Patient. An official American Thoracic Society workshop report: The Integrated Care of the COPD Patient. Proc Am Thorac Soc. 2012;9: 9-18. doi: [10.1513/pats.201201-014ST](https://doi.org/10.1513/pats.201201-014ST).

9. Spruit MA, Singh SJ, Garvey C, ZuWallack R, Nici L, Rochester C, et al. An official American Thoracic Society/European Respiratory Society statement: Key concepts and advances in pulmonary rehabilitation. Am J Respir Crit Care Med. 2013;188: e13-e64. doi: [10.1164/rccm.201309-1634ST](https://doi.org/10.1164/rccm.201309-1634ST).

10. Adams SG, Smith PK, Allan PF, Anzueto A, Pugh JA, Cornell JE. Systematic review of the chronic care model in chronic obstructive pulmonary disease prevention and management. Arch Intern Med. 2007;167: 551-561. doi: [10.1001/archinte.167.6.551](https://doi.org/10.1001/archinte.167.6.551).

11. Berkhof FF, Hesselink AM, Vaessen DL, Uil SM, Kerstjens HA, van den Berg JW. The effect of an outpatient care on-demand-system on health status and costs in patients with COPD: A randomized trial. Respir Med. 2014;108: 1163-1170. doi: [10.1016/j.rmed.2014.05.011](https://doi.org/10.1016/j.rmed.2014.05.011).

12. Bentley CL, Mountain GA, Thompson J, Fitzsimmons DA, Lowrie K, Parker SG, et al. A pilot randomised controlled trial of a telehealth intervention in patients with chronic obstructive pulmonary disease: Challenges of clinician-led data collection. Trials. 2014;15: 313. doi: [10.1186/1745-6215-15-313](https://doi.org/10.1186/1745-6215-15-313).

13. Shwamm LH, Holloway RG, Amarenco P, Audebert HJ, Bakas T, Chumbler NR, et al. A review of the evidence for the use of telemedicine within stroke systems of care: A scientific statement from the American Heart Association/American Stroke Association, Stroke. 2009;40: 2616-2634.

14. Thijssing L, van der Heijden JP, Melissant CF, Chavannes NH, Witkamp L, Jaspers MW. Telepulmonology and telespirometry. InMIE. 2014;205: 211-215.

15. León A, Cáceres C, Fernández E, Chausa P, Martin M, Codina C, et al. A new multidisciplinary home care telemedicine system to monitor stable chronic human immunodeficiency virus-infected patients: A randomized study. PLOS ONE. 2011;6: e14515. doi: [10.1371/journal.pone.0014515](https://doi.org/10.1371/journal.pone.0014515).

16. Kamei T, Yamamoto Y, Kajii F, et al. Effectiveness of telenursing practice based on home monitoring to prevent acute exacerbation and rehospitalization of COPD home oxygen therapy patients: A randomized controlled trial to evaluate nursing skills. J Jpn Acad Nurs Sci. 2011;31: 24-33.

17. Azuma M. Influence of tele-nursing intervention on self-management behavior of type 2 diabetes patients. J Jpn Soc Telemed. 2012;8: 158-161.

18. Kikuchi H, Terui R, Kakiyama K, et al. Construction of a community-based tele-nursing system in a wide-area cold and snowy region: Effects on home care patients. J Jpn Soc Telemed. 2013;9: 159-162.

19. Berkhof FF, van den Berg JW, Uil SM, Kerstjens HA. Telemedicine, the effect of nurse-initiated telephone follow up, on health status and health-care utilization in COPD patients: A randomized trial. Respirology. 2015;20: 279-285. doi: [10.1111/resp.12437](https://doi.org/10.1111/resp.12437).

20. Regional communications promotion division; 2011: Telemedicine Model Reference Manual, viewed. [Cited December 5, 2016]. Available from: http://www.soumu.go.jp/main_content/000127781.pdf. Bureau: Information Distribution Administration, Ministry of Internal Affairs and Communications.

21. Japan Respiratory Society Special Committee on Lung Physiology and Japan Respiratory Management Society Oxygen Therapy Guideline Development Committee 2006, Oxygen Therapy Guideline. Medical view Inc.

22. WHO. World health statistics 2008; 2008.

23. Sugiyama K, Kadota J Gemma A. Respiratory diseases latest treatment 2016-2018, Nankendo; 2016.

24. The Japanese Respiratory Society White Paper on Home Respiratory Care Preparation Committee 2013. White paper on home respiratory care: COPD patient questionnaire survey disease-specific analysis, medical view-sha.

25. The Japanese Respiratory Society NPPV Guideline Development Committee. NPPV guideline. rev. 2nd ed., Nankido; 2015.

26. Spruit MA, Singh SJ, Garvey C, et al. An official American Thoracic Society/European Respiratory Society statement: Key concepts and Am J Respir. Crit Care Med. 2014;189: 1570.

27. Shimoyama M, Furuse M. Self-care acquisition process of patients with chronic respiratory failure undergoing home noninvasive positive pressure ventilation therapy. J Jpn Soc Nurs Res. 2012.

28. The Japanese Respiratory Society. COPD Guideline. Preparation Committee, Guidelines for COPD diagnosis and treatment. 4th ed., Medical Review Co; 2013.

29. de Marco R, Accordini S, Marcon A, Cerveri I, Antó JM, Gislason T, et al. Risk factors for chronic obstructive pulmonary disease in a European cohort of young adults. Am J Respir Crit Care Med. 2011;183: 891-897. doi: [10.1164/rccm.201007-1125OC](https://doi.org/10.1164/rccm.201007-1125OC).

30. Pulmonary Rehabilitation Committee of the Japanese Society for Pulmonary Care and Rehabilitation, et al. Pulmonary rehabilitation manual: Concept and practice of patient education, Terurinsha; 2007.

31. Hurst JR, Vestbo J, Anzueto A, Locantore N, Müllerova H, Tal-Singer R, et al. Susceptibility to exacerbation in chronic obstructive pulmonary disease. N Engl J Med. 2010;363: 1128-1138. doi: [10.1056/NEJMoa0909883](https://doi.org/10.1056/NEJMoa0909883).

32. Harrison SL, Janaudis-Ferreira T, Brooks D, Desveaux L, Goldstein RS. Self-management following an acute exacerbation of COPD: A systematic review. Chest. 2015;147: 646-661. doi: [10.1378/chest.14-1658](https://doi.org/10.1378/chest.14-1658).

33. Gosselink R, De Vos J, van den Heuvel SP, Segers J, Decramer M, Kwakkel G. Impact of inspiratory muscle training in patients with COPD: What is the evidence? Eur Respir J. 2011;37: 416-425. doi: [10.1183/09031936.00031810](https://doi.org/10.1183/09031936.00031810).

34. Rice K, Bourbeau J, MacDonald R, Wilt TJ. Collaborative self-management and behavioral change. Clin Chest Med. 2014;35: 337-351. doi: [10.1016/j.ccm.2014.02.004](https://doi.org/10.1016/j.ccm.2014.02.004).

35. Jefferson T, Foxlee R, Del Mar C, et al. Physical interventions to interrupt or reduce the spread of respiratory viruses: Systematic review. BMJ. 2008;336: 77-80. doi: 10.1136/bmj.39393.510347.BE.

36. Song HY, Yong SJ, Hur HK. Effectiveness of a brief self-care support intervention for pulmonary rehabilitation among the elderly patients with chronic obstructive pulmonary disease in Korea. Rehabil Nurs. 2014;39: 147-156. doi: [10.1002/rnj.92](https://doi.org/10.1002/rnj.92).

37. Mitsuzuka Y, Takahashi S, Iida S, et al. Problems in action plans for COPD exacerbations and effectiveness of telephone guidance. Japanese Society for Pulmonary Care and Rehabilitation, Vol. 25; 2015.

38. Gadoury MA, Schwartzman K, Rouleau M, Maltais F, Julien M, Beaupré A, et al. Self-management reduces both short- and long-term hospitalisation in COPD. Eur Respir J. 2005;26: 853-857. doi: [10.1183/09031936.05.00093204](https://doi.org/10.1183/09031936.05.00093204).

39. Mendoza L, Horta P, Espinoza J, Aguilera M, Balmaceda N, Castro A, et al. Pedometers to enhance physical activity in COPD: A randomised controlled trial. Eur Respir J. 2015;45: 347-354. doi: [10.1183/09031936.00084514](https://doi.org/10.1183/09031936.00084514).

40. Ueki J. Pulmonary rehabilitation and physical activity -Current status and issues regarding improvement and maintenance of physical activity-, Japanese. Respir Med. 2015;4: 36-40.

41. Jones PW, Quirk FH, Baveystock CM. The St George's Respiratory Questionnaire. Respir Med. 1991;85;Suppl B: 25–31; discussion 33. doi: [10.1016/s0954-6111(06)80166-6](https://doi.org/10.1016/s0954-6111(06)80166-6).

42. Nishimura K, Tsukino M, Hajiro T. Health-related quality of life in patients with chronic obstructive pulmonary disease. Curr Opin Pulm Med. 1998;4: 107-115. doi: [10.1097/00063198-199803000-00009](https://doi.org/10.1097/00063198-199803000-00009).

43. Hajiro T, Nishimura K, Tsukino M, Ikeda A, Koyama H, Izumi T. Comparison of discriminative properties among disease-specific questionnaires for measuring health-related quality of life in patients with chronic obstructive pulmonary disease. Am J Respir Crit Care Med. 1998;157: 785-790. doi: [10.1164/ajrccm.157.3.9703055](https://doi.org/10.1164/ajrccm.157.3.9703055).

44. Nishimura M, Makita H. Series: Clinical study from Japan and its reflections; Hokkaido COPD Cohort Study. Nihon Naika Gakkai Zasshi. 2013;102: 463–470. doi: 10.2169/naika.102.463’

# Appendix

1. Explanation and Consent Document
2. Case Report Form
3. Linkage Anonymization Table
4. Questionnaire
