# Supplementary material for: Effectiveness of a telenursing intervention program in reducing exacerbations in patients with chronic respiratory failure receiving noninvasive positive pressure ventilation: A randomized controlled trial
Source: PLoS One. 2023 Oct 26;18(10):e0269753. doi: 10.1371/journal.pone.0269753 (PMC10602241; doi:10.1371/journal.pone.0269753)
Supplement: S2 File — (DOC) [file pone.0269753.s008.doc]

第6版　2017年1月10日

| **第Ⅲ種**  **非侵襲的陽圧換気療法を受けている慢性呼吸不全患者の**  **急性増悪予防を目的とした遠隔看護介入プログラムの効果**  （臨床試験登録番号：UMIN000027657）  研究責任者  教授　佐藤 冨美子  東北大学大学院医学系研究科保健学専攻家族支援看護学講座がん看護学分野  〒980–8575  仙台市青葉区星陵町２−１  TEL　022−717−7926 FAX　022−717−7910  E-mail　fsato@med.tohoku.ac.jp  研究事務局  博士後期課程　霜山 真  東北大学大学院医学系研究科保健学専攻家族支援看護学講座がん看護学分野  〒980–8575  仙台市青葉区星陵町２−１  TEL　090−7798−1240  E-mail　makoto.shimoyama.r7@dc.tohoku.ac.jp  2017年7月21日 作成（第6版） |
| --- |

目次

[0. 概要 1](#__RefHeading___Toc477288358)

[1. 目的 3](#__RefHeading___Toc477288359)

[2. 背景と研究計画の根拠 3](#__RefHeading___Toc477288360)

[2.1. 背景 3](#__RefHeading___Toc477288361)

[2.2. 研究の科学的合理性の根拠 5](#__RefHeading___Toc477288362)

[3. 研究対象者の選定方針 6](#__RefHeading___Toc477288363)

[3.1. 適格基準 6](#__RefHeading___Toc477288364)

[3.2. 除外基準 6](#__RefHeading___Toc477288365)

[4. 研究の方法、期間 6](#__RefHeading___Toc477288366)

[4.1. 研究デザイン 6](#__RefHeading___Toc477288367)

[4.2. 観察の内容 7](#__RefHeading___Toc477288368)

[4.3. 併用療法 8](#__RefHeading___Toc477288369)

[4.4. 検査スケジュール 8](#__RefHeading___Toc477288370)

[4.5. 研究期間 8](#__RefHeading___Toc477288371)

[5. 有害事象の評価 8](#__RefHeading___Toc477288372)

[5.1. 情報の入手 8](#__RefHeading___Toc477288373)

[5.2. 有害事象の記載 10](#__RefHeading___Toc477288374)

[6. 重篤な有害事象／不具合発生時の対応（研究機関の長に報告する有害事象範囲を含む） 10](#__RefHeading___Toc477288375)

[6.1. 有害事象／不具合発生時の対応 10](#__RefHeading___Toc477288376)

[6.2. 研究機関の長、研究責任者（研究代表者）への報告 10](#__RefHeading___Toc477288377)

[6.3. 共同研究機関への報告 11](#__RefHeading___Toc477288378)

[7. 評価項目・方法 11](#__RefHeading___Toc477288379)

[7.1. 主要評価項目 11](#__RefHeading___Toc477288380)

[7.2. 副次的評価項目 11](#__RefHeading___Toc477288381)

[7.3. 評価の中央判定 12](#__RefHeading___Toc477288382)

[8. 登録・割付 12](#__RefHeading___Toc477288383)

[8.1. 登録 12](#__RefHeading___Toc477288384)

[8.2. 割付 12](#__RefHeading___Toc477288385)

[9. 予定症例数、設定根拠 12](#__RefHeading___Toc477288386)

[9.1. 予定症例数 12](#__RefHeading___Toc477288387)

[9.2. 設定根拠 12](#__RefHeading___Toc477288388)

[10. 統計解析 12](#__RefHeading___Toc477288389)

[10.1. 統計解析の方法 12](#__RefHeading___Toc477288390)

[10.2. 中間解析と研究の早期中止 13](#__RefHeading___Toc477288391)

[11. データの管理方法、自己点検の方法 13](#__RefHeading___Toc477288392)

[11.1. 症例記録（Case Report Form：CRF）の作成 13](#__RefHeading___Toc477288393)

[11.2. CRFの自己点検 13](#__RefHeading___Toc477288394)

[11.3. CRFの送付及び保管 13](#__RefHeading___Toc477288395)

[11.4. CRFの修正手順 13](#__RefHeading___Toc477288396)

[12. インフォームド・コンセントを受ける手続 14](#__RefHeading___Toc477288397)

[12.1. 研究対象者への説明 14](#__RefHeading___Toc477288398)

[12.2. 同意 14](#__RefHeading___Toc477288399)

[13. 代筆による同意取得の手続き 14](#__RefHeading___Toc477288400)

[14. 個人情報等の取扱い 15](#__RefHeading___Toc477288401)

[14.1. 個人情報の利用目的 15](#__RefHeading___Toc477288402)

[14.2. 利用方法（匿名化の方法） 15](#__RefHeading___Toc477288403)

[14.3. 安全管理責任体制（個人情報の安全管理措置） 15](#__RefHeading___Toc477288404)

[15. 研究対象者に生じる負担、予測されるリスク（起こりうる有害事象を含む）・利益、これらの総合的評価、負担・リスクを最小化する対策 15](#__RefHeading___Toc477288405)

[15.1. 研究参加に伴って予測される利益と不利益の要約 15](#__RefHeading___Toc477288406)

[16. 試料・情報等の保存・廃棄の方法 16](#__RefHeading___Toc477288407)

[16.1. 保存 16](#__RefHeading___Toc477288408)

[16.2. 廃棄 16](#__RefHeading___Toc477288409)

[17. 研究の資金源等、研究機関の研究に係る利益相反及び個人の収益等、研究者等の研究に係る利益相反に関する状況 16](#__RefHeading___Toc477288410)

[18. 知的財産 16](#__RefHeading___Toc477288411)

[19. 研究に関する情報公開の方法 16](#__RefHeading___Toc477288412)

[19.1. 研究計画の登録 16](#__RefHeading___Toc477288413)

[19.2. 研究結果の登録 16](#__RefHeading___Toc477288414)

[19.3. 研究結果の公表 16](#__RefHeading___Toc477288415)

[20. 研究機関の長への報告内容、方法 17](#__RefHeading___Toc477288416)

[21. 研究対象者等、その関係者からの相談等への対応 17](#__RefHeading___Toc477288417)

[22. 研究対象者等に経済的負担または謝礼がある場合、その旨、その内容 17](#__RefHeading___Toc477288418)

[23. 侵襲を伴う研究の場合、研究によって生じた健康被害に対する補償の有無、内容 17](#__RefHeading___Toc477288419)

[24. 試料・情報が同意を受ける時点では特定されない将来の研究のために用いられる可能性／他の研究機関に提供する可能性がある場合、その旨と同意を受ける時点において想定される内容 17](#__RefHeading___Toc477288420)

[25. 本研究の共同研究機関、あるいはそれ以外の研究機関への試料・情報等の提供 17](#__RefHeading___Toc477288421)

[26. 営利団体等への試料・情報等の提供 17](#__RefHeading___Toc477288422)

[27. 研究計画書の変更 18](#__RefHeading___Toc477288423)

[28. 研究の実施体制 18](#__RefHeading___Toc477288424)

[28.1. 研究機関の名称、研究責任者の氏名 18](#__RefHeading___Toc477288425)

[28.2. 共同研究機関 18](#__RefHeading___Toc477288426)

[28.3. 研究事務局、研究者の役割 19](#__RefHeading___Toc477288427)

[28.4. 統計解析、データセンター 20](#__RefHeading___Toc477288428)

[28.5. 研究に関する問合せ窓口 20](#__RefHeading___Toc477288429)

[29. 引用文献 20](#__RefHeading___Toc477288430)

[30. Appendix 22](#__RefHeading___Toc477288431)

# 概要

- 1. **シェーマ**

|  |  | 外来通院中のNPPVを受けている慢性呼吸不全患者 | | | | | |  |  |
| --- | --- | --- | --- | --- | --- | --- | --- | --- | --- |
|  |  |  |  |  |  |  |  |  |  |
|  | 定期外来受診時、主治医または担当医より研究の概要を説明し、研究参加意思あり | | | | | | | |  |
|  |  |  |  |  |  |  |  |  |  |
|  |  | 研究者により再度、研究概要を説明、同意書を記入し参加登録 | | | | | |  |  |
|  |  |  |  |  |  |  |  |  |  |
|  | 参加登録時（ベースライン）  【主要評価項目】過去3カ月間の入院回数、入院日数、定期外外来受診回数の確認  【副次評価項目】SGRQ、EQ-5D、SCAQ回答、呼吸機能検査、6分間歩行試験 | | | | | | | |  |
|  |  |
|  |  |
|  |  |  |  |  |  |  |  |  |  |
|  |  |  | 割付（割付調整因子：施設） | | | |  |  |  |
|  |  |  |  |  |  |  |  |  |  |
|  |  |  |  |  |  |  |  |  |  |
| 介入群：  通常の外来診療+遠隔看護介入プログラム | | | |  |  | 対照群：  通常の外来診療 | | | |
|  |  |
|  |  |  |  |  |  |  |  |  |  |
| 通常の外来診療（１回/月）  ・担当医による問診、診察、処方  ・外来看護師による健康相談・情報提供  遠隔看護介入プログラム（１回/日・3カ月）  1.遠隔モニタリング  ・１日１回　タブレット端末への入力データの観察を実施  ・入力項目：バイタルサイン、療養生活上の呼吸器症状、食事摂取状況、排泄状況、服薬状況、呼吸器以外の身体症状  2.健康相談・情報提供  ・遠隔モニタリングの結果、トリガー該当時（急性増悪の徴候出現時）または対象者の希望時に１日１回30分程度 テレビ電話による健康相談・情報提供を実施 | | | |  |  | 通常の外来診療（１回/月）  ・担当医による問診、診察、処方  ・外来看護師による健康相談・情報提供 | | | |
|  |  |
|  |  |
|  |  |
|  |  |
|  |  |
|  |  |
|  |  |
|  |  |
|  |  |
|  |  |
|  |  |  |  |  |  |
|  |  |  |  |  |  |
|  |  |  |  |  |  |
|  |  |  |  |  |  |
|  |  |  |  |  |  |
|  |  |  |  |  |  |  |  |  |  |
|  |  |  |  |  |  |  |  |  |  |
|  | 参加登録から3カ月後  【主要評価項目】過去3カ月間の入院回数、入院日数、定期外外来受診回数の確認  【副次評価項目】SGRQ、EQ-5D、SCAQ回答、呼吸機能検査、6分間歩行試験 | | | | | | | |  |
|  |  |
|  |  |
|  |  |  |  |  |  |  |  |  |  |
|  | 【介入により期待される効果】  　遠隔看護介入プログラムを行うことにより、介入群のセルフマネジメント行動の継続が期待され、主要評価項目と副次評価項目が対照群と比較して有意に改善する | | | | | | | |  |
|  |  |
|  |  |

- 1. **目的**

本研究の目的は、在宅療養において非侵襲的陽圧換気療法（Noninvasive Positive Pressure Ventilation：以下NPPV）を受けている慢性呼吸不全患者への情報通信技術（Information and Communication Technology：以下ICT）を活用した遠隔看護介入プログラムによる急性増悪予防効果を無作為化比較試験で検証する。

- 1. **対象**

研究協力の同意を得た東北大学病院および協力機関の呼吸器内科外来に通院中のNPPVを受けている慢性呼吸不全患者とする。適格基準は①呼吸器疾患を原疾患とする者、②認知機能障害がない者、③外来通院できる者、④20歳以上の者とする。

- 1. **予定症例数、研究期間**

1. 予定症例数：

先行文献より急性増悪によって救急外来を受診したNPPV実施者の平均回数は2.4回/年、NPPV非実施者の受診回数1.6回/年を参考に1.0回/年の改善をはかると仮定する。有意水準5％、検出力80％、改善すると仮定した受診回数の標準偏差を1.0回/年で算出して、介入群・対照群を各15名とする。また、研究協力の同意をしていても研究途中での脱落者の割合を全体の10％と見込んで算定を行い、介入群・対照群を各17名とする。したがって、本研究の予定症例数は34例とする。

1. 研究期間：倫理委員会承認後（2017年5月）～2019年3月

登録期間：倫理委員会承認後～2018年10月

- 1. **問合せ先**

1. 適格基準、治療変更基準等、臨床的判断を要するもの、登録手順、記録用紙（CRF）記入等

研究事務局

博士後期課程　霜山 真

東北大学大学院医学系研究科保健学専攻家族支援看護学講座がん看護学分野

TEL　090−7798−1240

E-mail　makoto.shimoyama.r7@dc.tohoku.ac.jp

# 目的

在宅療養においてNPPVを受けている慢性呼吸不全患者に対するICTを活用した遠隔看護介入プログラムの有効性について、急性増悪による再入院と臨時受診回数、再入院率と臨時受診率を主要評価項目として検討する。

# 背景と研究計画の根拠

# 背景

近年、我が国では諸外国に例を見ない速度で高齢化が進行しており、団塊世代が75歳以上となる2025年までに、要介護状態でも住み慣れた地域で自分らしい生活を人生の最後まで続けることができるよう地域包括ケアシステムの構築が求められている^1）-3）^。また、2025年までに約30万床が病院病床から在宅医療へ移行することが計画されており^4）^、病院完結型医療から地域完結型医療への転換が加速的に進められている^5）^。地域包括ケアシステムの運用を支える手段の一つとして、ICTを活用した遠隔医療が挙げられる^6）^。我が国の遠隔医療では、ICTが新たな富の創出や生産活動の効率化に大きく貢献し、国民生活を便利にするものと考えられており、超高齢化社会に向けてICTを活用する方策を検討していくことを明言している^7）^。米国胸部疾患学会と欧州呼吸器学会は慢性呼吸器疾患患者への統合ケアを推奨しており^8）9）^、「適切な時に適切な場所で患者の個々のニーズにあった適切なケアの提供」を原則としている^10)^。欧米ではこの原則に基づき、遠隔医療が著しく普及し、対象者がどこに居住していても等質な医療や看護援助の提供を受け、在宅医療費の削減に効果を上げている^11)^。一方で我が国における遠隔医療は1970年代に始まり、その後、遠方の医師と医師との間で行われる遠隔画像診断や遠隔術中病理診断、遠方の医師と患者間で行われる遠隔診療^12)-15)^、看護師が患者との間で健康相談を行う遠隔看護など^16)-18)^により、遠隔医療の基盤が構築されつつある。特に遠隔看護は健康管理を必要とする患者の健康増進を目的に生体データを収集し、健康状態の把握とともに的確な健康相談や健康指導の機会を提供することが可能となる^19)^。また、患者とのテレビ電話での画像や音声による双方向のコミュニケーションにより、患者の抱く不安を解消することを可能とし、質の高い健康管理を提供することができるため^20)^、遠隔看護の実践が徐々に広まりつつある。

慢性呼吸不全は1か月以上持続する呼吸不全と定義される^21)^。慢性呼吸不全を呈する代表的な疾患は慢性閉塞性肺疾患（Chronic Obstructive Pulmonary Disease：以下COPD）であり、慢性呼吸不全患者の45％を占める^22)^。2008年の世界保健機関（World Health Organization：以下WHO）の報告^23)^によると、2030年にはCOPDが死因の第3位になると予測し、今後、慢性呼吸不全患者の増加が推察される。在宅呼吸ケア白書によると、外来COPD患者の多くが在宅酸素療法（Home Oxygen Therapy：以下HOT）やNPPVを受けており^24)^、慢性呼吸不全患者を取り巻く在宅医療の高度化を示している。また、非薬物療法として呼吸リハビリテーション、日常生活動作の工夫などの患者教育等が中心となり、原因疾患に関係なく共通した治療方針が示されている^23)^。米国胸部疾患学会と欧州呼吸器学会から、呼吸リハビリテーションは「徹底した患者のアセスメントに基づいた包括的な医療介入に引き続いて、運動療法、教育、行動変容だけでなく慢性呼吸器疾患患者の身体および心理的な状況を改善し、長期の健康増進に対する行動のアドヒアランスを促進するために患者個々の必要性に応じた治療が行われるもの」と定義しており^9)^、オーダーメイドの非薬物療法による介入の必要性を示唆している。NPPVは鼻口マスクをインターフェースとし、慢性呼吸不全に対する換気補助療法の一つとして、主に睡眠中の使用で動脈血液ガス、呼吸困難、朝方の頭痛や倦怠感などの症状を改善する。気管挿管を必要としないため、会話や飲食等に支障をきたさず、日常生活を送ることができる。慢性呼吸不全患者へのNPPV導入により、患者の生命予後や著しくQuality of Life（以下QOL）が改善している^25）^。しかしながら、慢性呼吸不全のⅡ型呼吸不全はガス交換障害による低酸素血症だけではなく、換気不全による高二酸化炭素血症を併存している^21)^。また、NPPVを受けている慢性呼吸不全患者は呼吸器症状による活動範囲の減少やマスク装着時の苦痛を経験し、その経験がNPPV機器活用の阻害因子となりCO_2_ナルコーシスの危険性を高くする^27）^。Ⅱ型呼吸不全患者はⅠ型呼吸不全患者と同じような日常生活を送っているように捉えられるが、低酸素状態に加え高二酸化炭素状態に陥りやすく、より複雑な身体状態の管理を身に付ける必要がある。患者自らが急性増悪時の呼吸困難や息切れに対処しようとして安易に酸素量を増加させることは高二酸化炭素状態を惹起させ、CO_2_ナルコーシスの引き金となることが推察される。呼吸器症状出現時の適切な対処行動や知識を身に付けることが不用意なCO_2_ナルコーシスの機会を減らすこととなる。加えて、NPPVを受けている慢性呼吸不全患者は呼吸機能の予備能や運動耐用能も乏しいことから感冒などにより容易に急性増悪をきたす^23)^。それを契機として心不全などの全身状態の悪化を引き起こし即座に致命的な状況となる。慢性呼吸不全患者は平素から咳嗽や喀痰、呼吸困難などの呼吸器症状があるため、急性増悪の変化に気づきにくく医療機関への受診が遅れてしまうため、より重篤な状況に陥る可能性がある。入院の長期化や度重なる入院は日常生活動作（Activities of Daily Living：以下ADL）の低下をもたらし、QOL低下の要因となる^25)^。

慢性呼吸不全患者の急性増悪とは呼吸困難、咳嗽、喀痰などの呼吸器症状ならびに呼吸不全状態が急激に悪化することと定義されており、呼吸器感染症、大気汚染、気胸、疲労による右心負荷、心理的要因、睡眠薬の過剰投与、不適切な酸素投与、原疾患の悪化などが加わったときに急性増悪を起こす可能性がある^23)^。慢性呼吸不全患者の急性増悪の原因を詳細に述べている文献は乏しい。慢性呼吸不全の原因疾患の一つであるCOPDガイドラインによると、急性増悪の原因は50～70％が感冒をはじめとする呼吸器感染症、10％が大気汚染、約30％が原因不明である^28)^。呼吸器感染症では、常在菌のインフルエンザ菌、肺炎球菌、ウイルス感染ではインフルエンザウイルス、アデノウイルスなどが原因となる。大気汚染物質はオゾン、窒素酸化物、大気中の直径10μm以下の浮遊粒子状物質などの吸入が原因とされる。特にたばこ煙は約4000種類の化学物質、約200種類の有害物質、60種類以上の発がん物質が含まれており、2.5μm以下の微粒子となるため、急性増悪の原因となる環境を作り出す^29)^。そのため、慢性呼吸不全患者の生活には禁煙環境の徹底が求められる^30)^。このように慢性呼吸不全患者の急性増悪への対策には、呼吸器感染の予防や大気汚染環境の回避が重要となる。呼吸器感染の予防には、身体の抵抗力向上のため適切な休養や睡眠の確保、栄養管理が必要である^23）30）^。また、局所的なウイルスや細菌を除去する方法としてマスクの着用、手洗いやうがいの励行、身体の清潔保持、特異的な予防手段として各種ワクチンの接種^31)^がある。他にも適度な保温や加湿といった環境調整や呼吸リハビリテーションなどの身体活動の維持、薬物治療の管理が急性増悪への対策として効果的であるとの報告がみられる^32)-35)^。一方で、呼吸困難は生命維持の不安や恐怖を生じ、身体活動量の減少や筋力低下を引き起こし、呼吸困難を助長させるという悪循環を引き起こすことが報告されている^27)^。慢性呼吸不全患者は療養生活を送る上で医療者に対して、「息切れを軽くする日常生活の工夫」や「呼吸訓練」などの療養生活に関する情報を望んでいるとの報告があり^24)^、不安を抱えながら療養生活を送っている現状が推察される。Samanthaらは、慢性呼吸不全患者が呼吸器症状に応じた対処行動や安定期を長く過ごすための感染予防、栄養管理、呼吸リハビリテーションといった知識や技術を活用し、セルフマネジメントを効果的に行うことで急性増悪の回避に結びつくことを報告している^32)^。

臨床現場では在院日数の短縮化や在宅療養の推進、マンパワー不足から、医療者は限られた時間的制約の中で治療や検査を行っているため、個別性に配慮した情報提供や健康相談は不足していることが推察される。現在、在院時には症状改善のための薬物療法、知識獲得のための情報提供や身体耐用能の維持向上のための呼吸訓練などの非薬物療法が実施され、外来診療時にも継続されている。しかし、それらの効果は在院時のみならず自宅での療養生活で継続できるか否かによって、呼吸器症状の経過に影響を及ぼすことが報告されている^36)^。そのため、慢性呼吸不全患者は在宅療養における治療に対して患者自身が理解し、納得した上で主体的にセルフマネジメントを実践し、日常生活を営んでいくことで急性増悪予防やQOLが維持される。これまでに国内外において、看護師によるHOTを受けている慢性呼吸不全患者を対象とした呼吸リハビリテーションやアクションプランの導入の実践報告^37)-39)^、テレナーシング等の遠隔医療が急性増悪防止や身体活動量の向上に効果的であることが明らかにされている^16)^。しかし、より医療依存度の高い状態であるNPPVを受ける慢性呼吸不全患者を対象とした遠隔看護は、急性増悪予防やQOL維持のために非常に重要な支援であると考えられるが、いまだに確固たるエビデンスの立証には至っていない。

そこで、本研究はNPPVを受けている慢性呼吸不全患者が心身の状態に応じて、主体的に症状マネジメントや感染予防行動などの非薬物療法を組み込んだ生活を支援する遠隔看護介入プログラムの効果を検証することを目的とする。現在、NPPVを受けている慢性呼吸不全患者の平均年齢は72.6歳であり、全体の8割が60歳以上の高齢者である^24)^。患者の高齢化から医療的介入のみならず社会的支援も必要であると考えられる。しかしながら、介護認定基準の中に直接的に呼吸器障害を判定する項目はなく、慢性呼吸不全患者は無理をすれば動けることから、他の身体障害等を有する者に比べ介護認定が低くなりがちであり、必要とする介護支援が十分に増やしにくいという問題点がある。高齢化に伴う視力低下や認知力の低下が考えられ、適切にセルフマネジメントを行うためには、より簡便に身体症状の変化を明確に示すことができ、把握した身体状態に合わせた生活支援ツールが必要となる^40)^。現在、在宅療養中の慢性呼吸不全患者は療養日誌などを記帳し、自身の体調管理に勤めているが、より治療効果を自覚できるように身体活動量やバイタルサイン等の身体状況を可視化しながら、遠隔的に患者指導や健康相談を行う電子ツール、患者が知識と技術の習得やセルフモニタリングがより容易となる教育ツールが必要である。したがって、患者が日々の身体状況の可視化を可能とする電子療養日誌を作成、電子療養日誌を用い個別性に合った生活指導を遠隔看護介入プログラムに含む。電子療養日誌で得られた情報を患者と看護師で共有し、療養生活上で生じた不安感や恐怖感の緩和するために、必要時にソーシャルネットワーキングサービス（Social Networking Service：以下SNS）機能を用いて意思を伝え、テレビ通話による健康相談を行うことが遠隔看護介入プログラムには必要である。本研究により、NPPVを受けている慢性呼吸不全患者が自宅で安全にセルフマネジメントを実施することが可能となり、呼吸困難感の軽減や健康関連QOLの改善をもたらし、急性増悪による再入院回数および臨時外来受診回数、再入院率および臨時受診率の減少をはかる。

# 研究の科学的合理性の根拠

慢性呼吸不全は肺や気道といった呼吸器の不可逆的な変化により生じた呼吸不全が1カ月以上持続する状態であり、その病態によりⅠ型呼吸不全とⅡ型呼吸不全に分類することができる。Ⅰ型呼吸不全に対する治療として酸素療法、Ⅱ型呼吸不全に対して酸素療法に加え換気補助療法が必要となる。近年の医療の発展とともに、在宅療養における酸素療法や補助換気療法の普及が著しくQOL維持向上には欠かすことのできない治療と言える。慢性呼吸不全に陥った患者は呼吸予備能が乏しく急性増悪をきたしやすい状態であり、医療機器の管理とともに退院後の呼吸リハビリテーションやセルフマネジメントの継続はQOL向上には必要不可欠な問題である。慢性呼吸不全患者は主な症状として呼吸困難を抱えるがADLの障害まで生じないため、自らの健康管理により在宅療養を送っている。在宅療養中の慢性呼吸不全患者は増加傾向にあり、健康状態を維持するためにセルフモニタリングや呼吸困難への対処行動、感染予防行動などのセルフマネジメントの継続が急性増悪予防の鍵となる。本研究は慢性呼吸不全患者の身体状態をタブレット端末により可視化させ、自らの健康状態の管理を支援する。一方で遠隔モニタリングを行い、遠隔地より健康相談や情報提供を行う遠隔看護介入プログラムの開発を行い、急性増悪予防効果を検証する。慢性呼吸不全患者にとって、急性増悪を繰り返すことは身体活動量や呼吸予備能の低下を招きQOL低下を引き起こすため、遠隔看護介入プログラムの検証は喫緊の課題であり、慢性呼吸不全患者に対する医療には欠かすことのできない研究である。また、慢性呼吸不全患者にとって在宅療養生活が保たれることで、急性増悪予防や再入院防止等の医療費抑制の一助となることから本研究は経済的にも非常に意義の大きい研究であると考えられる。本研究を実施することの適否について、倫理的・科学的および医学的妥当性の観点から東北大学大学院医学系研究科倫理委員会による審査いただき、研究機関長による承認を得ることとする。

# 研究対象者の選定方針

# 適格基準

対象は東北大学大学院医学系研究科倫理委員会および各協力施設倫理委員会の承認後から平成31年3月までに、研究協力の同意を得た東北大学病院呼吸器内科外来に通院中のNPPVを受けている慢性呼吸不全患者とする。以下の適格基準をすべて満たす患者を対象とする。

1. COPDや肺結核後遺症などの呼吸器疾患を原疾患とする慢性呼吸不全患者で、非侵襲的陽圧換気療法を受けている者とする。呼吸不全の期間が1か月以上持続しており、動脈血二酸化炭素分圧が45mmHg以上となるⅡ型呼吸不全状態にある者とする。
2. 本研究では疾患を特定しないため、病期やステージは設定することができない。
3. 登録時の年齢は20歳以上とし、タブレット端末を扱える者であれば対象とするため、年齢の上限は設定しない。
4. 本研究は性別によって、介入が変化することは考えていないため、性別は問わない。

# 除外基準

除外基準は、①認知機能障害によりコミュニケーションがとれない者、②日本語が話せない者、③外来通院できない者、④20歳未満の者とする。

認知機能障害が生じていることでタブレット端末の操作、意思疎通がはかれない可能性があるため、除外基準として設定する。認知機能障害と同様に日本語が話すことができないと研究者との意思疎通がはかれず、評価に影響を及ぼすと考えた。また、外来通院できない状態は、ADLが低下しており、自らでセルフケアや対処行動をうまく行うことができない状態であり、評価に影響を及ぼすと判断した。

# 研究の方法、期間

# 研究デザイン

1. **研究デザイン**

　通常診療に加え在宅療養においてNPPVを受けている慢性呼吸不全患者に対するICTを活用した遠隔看護介入プログラムを用いる介入群と通常診療を行う対照群を設定した無作為化比較試験である。

1. **研究デザインの設定根拠**

本研究はNPPVを受けている慢性呼吸不全患者を対象とした遠隔看護介入プログラムの有効性を検討することを目的としている。そのため、遠隔看護介入プログラムを行う患者群（介入群）は介入期間の急性増悪による臨時外来受診（受診割合、回数）が通常の診療のみの患者群（対照群）と比較して少ないことを仮説とし、本研究デザインを無作為化比較試験として設定した。

# 本研究で行う遠隔看護介入プログラムの概要と観察の内容

NPPVを受けている慢性呼吸不全患者の急性増悪予防を目的とした遠隔看護介入プログラムは、研究者が予備調査の結果と文献的考察に基づき開発した遠隔看護システムを用いて、NPPVを受けている慢性呼吸不全患者が個々の心身の状態に応じて、主体的にセルフモニタリングと症状への対処行動などのセルフマネジメントを生活に組み込み、急性増悪を予防する看護介入プログラムである。本プログラムは通常診療外の行為となるが診療に活用できる情報が含まれるため担当医と協働しながら実施する。実施内容は、主に①対象の日々の身体状態を遠隔地でモニタリング、②対象の日々の生活を送る上での疑問点や問題点に対する健康相談、呼吸器症状が軽減するための生活習慣やセルフマネジメントに関する情報提供である。本プログラムの構成は①NPPVを受けている慢性呼吸不全患者の自宅からのタブレット端末を用いたデータ入力、②研究者のパソコン端末からの遠隔モニタリングおよび健康相談、③データベースサーバー、④東北大学病院呼吸器内科主治医、⑤携帯電話通信から成り立つ。タブレット端末は研究者より貸与して提供される。遠隔看護方法は対象が1日1回、一定の時刻にタブレット端末に表示される質問項目に対して、10分程度の作業時間で選択肢の中から回答し、身体状況に関するデータをサーバーへ送信する。その内容はバイタルサイン、療養生活上の呼吸器症状、食事摂取状況、排泄状況、服薬状況、呼吸器以外の身体症状、医療者への質問内容とし、対象はタブレット端末画面からタッチパネル上のボタンによって回答する。患者がバイタルサイン等の測定情報をタブレットに入力後、送信されたデータを即時モニタリングし、事前に担当医と共に設定しておいた各質問項目のトリガーポイントと看護対応に基づき、トリアージを行う。トリガー該当の場合、設定しておいた看護対応に従い、電話やテレビ電話を用いて直接心身のアセスメントを行い、呼吸リハビリテーションマニュアル^30）^に基づき看護支援を行う。トリガー該当がなければ、メッセージ配信により終了となる。トリガー項目は全24項目である。トリガー該当項目の一つとして、経皮的酸素飽和度（SpO_2_）が挙げられる。SpO_2_が95％以上の場合、トリガー該当・看護対応はなしとする。SpO_2_が94％～90％の場合、トリガーを要注意と設定、他のデータと合わせて病状確認する。SpO_2_が89％以下の場合、トリガー該当と設定し、テレビ電話を用いた病状確認と看護対応を行う。対象に応じ個別に質問項目に対応した回答の閾値と看護対応を設定する。看護対応の例としては、SpO_2_が低下している原因を心身の状況からアセスメントし、原因に沿って服薬行動やNPPV装着、息切れ時の対応（呼吸法等）、感染予防行動などのセルフマネジメント行動が適切に行えるようにタブレット端末画面上のテレビ電話を用いた健康相談・情報提供を行う。看護対応後、担当医に対応内容を電話およびメールで報告し、翌日以降の対応を検討する。テレビ電話による看護対応を行い、状況判断に迷う場合には速やかに担当医に電話連絡し、対応を検討する。対象とのテレビ電話でのやり取りは、タブレット端末入力後のデータをもとに対象の希望時またはトリガー該当時に行われる。タブレット端末入力時間帯は8時～12時とし、看護対応は13時～17時に1日1回30分程度を想定している。タブレット端末への未入力が2日以上続いた際には速やかに対象へ電話連絡を行い、原因の解消をはかる。サーバーの設置は本システム制作会社に依頼し、送信データのSecure Sockets Layer（以下SSL）暗号化をはかり、サーバーへのアクセス権限は研究者および協力医療機関の担当医のみとする。通信技術として、NTTドコモと一般契約し、携帯電話通信網Long Term Evolution（以下LTE）サービスを用いる。独自のセキュリティ機能を用いて非常に堅牢な情報漏洩対策を行っているため、本研究には最適であると考えた。なお、タブレット端末は研究者からの貸与となるため、介入終了後に返却となる。

遠隔看護システム内のWeb上プログラムは患者サイトと研究者サイトから成り立つ。患者サイトのタブレット端末上は①日々の身体状態の記録画面、②テレビ通話画面、③SNS入力画面、④呼吸リハビリテーション情報の画面とする。研究者サイトは①対象の遠隔モニタリングデータ一覧、②コメント入力画面とする。研究者は対象の日々の記録を確認でき、経時変化をサマリーシートとして出力できる。

介入期間は24週間とし、主要評価項目の過去12週・24週間の臨時受診回数と再入院状況は参加登録時、参加登録から12週間後と24週間後に研究者が電子カルテから調査する。参加登録時、参加登録から12週間後と24週間後の定期外来受診時に、研究者が生活に関する質問紙調査と呼吸機能検査、6分間歩行試験を行う。また、参加登録から12週間後と24週間後に活動量計を確認し、平均歩数と活動量を収集する。本研究において実施される参加登録時、参加登録から12週間後と24週間後の呼吸機能検査や6分間歩行試験は通常診療外の検査となり保険診療として適さないため、研究費から支弁する機器を用いて研究者が実施する。本研究は急性増悪による臨時受診回数や再入院状況を主要評価項目としているため、急性増悪による症状の悪化において介入中止は設定しない。なんらかの出来事で対象が死亡した際には調査を継続できないため、介入を中止しなければならない。また、本プログラム参加後に継続希望があった場合、介入群・対照群に限らず、期間を延長して提供する可能性がある。

# 併用療法

1. **許容する併用療法**

　本研究の介入内容は主に遠隔モニタリング、情報提供や健康相談となる。これまでの治療を在宅療養生活の中でうまく継続していくことが重要となるため、他の治療によって本プログラムが障害されることはない。そのため、患者が行っている本プログラム以外の併用療法はすべて許容される。

1. **許容されない併用療法**

　許容されない併用療法は特に設定していない。

# 検査スケジュール

|  | 評価項目 | 調査スケジュール | | |
| --- | --- | --- | --- | --- |
|  |  | 参加登録時  （ベースライン） | 参加登録から12週後 | 参加登録から24週後 |
| 面接または診療録 | 患者基本情報 | ◯ |  |  |
|  | 過去12週間の臨時受診・再入院状況 | ◯ | ◯ |  |
|  | 過去24週間の臨時受診・再入院状況 | ◯ |  | ○ |
| 質問紙調査 | SGRQ | ◯ | ◯ | ◯ |
|  | EQ-5D | ◯ | ◯ | ◯ |
|  | SCAQ | ◯ | ◯ | ◯ |
| 客観的評価項目 | 呼吸機能検査 | ◯ | ◯ | ◯ |
|  | 6分間歩行試験 | ◯ | ◯ | ◯ |
|  | 平均歩数、活動量 |  | ◯ | ◯ |

# 研究期間

　研究期間は東北大学大学院医学系研究科倫理委員会承認後（2017年5月）から2019年3月までとする。

# 有害事象の評価

# 情報の入手

1. 研究者は重篤な有害事象／不具合が発現した場合、適切な処置を行い、研究機関の研究責任者に報告する。
2. 研究機関の研究責任者は、研究分担者に以下を確認する。

**研究**責任者による研究者等への確認事項

| ①有害事象名／不具合名  ②重症度分類^1)^  ③重篤性^2)^ 、重篤と判断した理由  ④予測性（未知・既知）^3)^  ⑤介入（試験薬／試験機器）との因果関係  ⑥事象／不具合の経緯（発現日、経過、転帰等）  ⑦被験者の特定に関する情報（イニシャル、年齢、性別） |
| --- |

**^1)^重症度分類**

National Cancer Institute Common Terminology Criteria for Adverse Events（NCI CTCAE v4.0：<http://www.jcog.jp/doctor/tool/CTCAEv4J_20150310.pdf>）等に従って判定する。

NCI CTCAE分類に該当する項目がない場合、以下**「有害事象の重症度分類基準」**を参考に判定する。

| **重症度分類**  **（NCI CTCAE Grade）** | **基準** |
| --- | --- |
| **軽症　（Grade1）** | 症状がない、または軽度の症状がある。臨床所見または検査所見のみ。  治療を要さない。 |
| **中等症　（Grade2）** | 最小限/局所的/非侵襲的治療を要する。  年齢相応の身の回り以外の日常生活動作の制限^*^。 |
| **重症　（Grade3）** | 重症または医学的に重要であるが、ただちに生命を脅かすものではない。  入院または入院期間の延長を要する。  活動不能/動作不能。身の回りの日常生活動作の制限^**^。 |
| **最重症　（Grade4）** | 生命を脅かす。緊急の処置を要する。 |
| **死亡　（Grade5）** | 有害事象（AE）による死亡。 |

^*^身の回り以外の日常生活動作（instrumental ADL）

：食事の準備、日用品や衣類の買い物、電話の使用、金銭の管理等。

^**^身の回りの日常生活動作（self care ADL）

：入浴、着衣・脱衣、食事の摂取、トイレの使用、薬の服薬が可能で、寝たきりではない状態。

**^2)^重篤の定義**

| ①死に至るもの  ②生命を脅かすもの  ③治療のための入院又は入院期間の延長が必要となるもの  ④永続的又は顕著な障害・機能不全に陥るもの  ⑤子孫に先天異常を来すもの |
| --- |

研究計画書で規定する入院、研究前（同意取得前）より予定していた療法または検査を研究実施中に実施することのみを目的とした入院（予定手術や検査等）、有害事象に伴う治療・検査の目的以外の入院（健康診断等）は重篤な有害事象として取扱わない。

**^3)^**予測性の定義

| **○予測できない（未知）**  当該事象等の発現、あるいは発現数、発現頻度、発現条件等の発現傾向が当該試験薬／試験機器に関する公式文書（添付文書や論文等）から予測できないもの  **○予測できる（既知）**  当該事象等の発現、あるいは発現数、発現頻度、発現条件等の発現傾向が当該試験薬／試験機器に関する公式文書（同上）から予測できるもの |
| --- |

# 有害事象の記載

本研究では、患者に対する情報提供や健康相談活動となるため、介入による有害事象は極めて少ないことが考えられる。しかしながら、副次評価項目の測定の際に呼吸機能検査や6分間歩行試験を行うため、一時的な症状の悪化などの軽微な有害事象につながってしまう可能性は否定できない。有害事象が発現した場合、速やかに主治医に連絡し対応を依頼する。また、すべての有害事象に関し、介入中の経過内容に、有害事象名、程度、重篤と判断した理由、発現日、転帰日、処置、転帰、介入との因果関係を含め、症例報告書に記載する。

# 重篤な有害事象／不具合発生時の対応（研究機関の長に報告する有害事象範囲を含む）

# 有害事象／不具合発生時の対応

1. 研究者は有害事象／不具合が発現した場合、適切な処置を施し、研究対象者の安全確保に留意して原因究明に努める。本研究は評価項目として6分間歩行試験を予定している。本研究における6分間歩行試験は診療外の検査であるため、呼吸不全増悪時に速やかに適切な医療が提供できるよう、事前に担当医や外来看護師などと体制を整備し安全性を高める。また、共同研究施設の担当医に対しても同様の対応が行えるように体制を整備し、対象の安全の確保に努める。
2. 研究者等は、発現した症状あるいは臨床検査値の異常変動について、原則として当該事象が消失または研究開始前の状態に回復するまで、または臨床上問題とならないと判断されるまで、可能な限り経過観察を継続し、その転帰を確認する。
3. 研究終了時に未回復の有害事象／不具合が非可逆的な事象の場合等、研究者等が追跡不要と判断した場合、研究対象者の研究終了時をもって追跡終了し、症例報告書のコメント欄に追跡不要と判断した理由を記載する。

# 研究機関の長、研究責任者（研究代表者）への報告

1. 研究機関の研究責任者は、重篤な有害事象／不具合の発現を知った時点から以下の期限内に研究機関の長に報告する。報告は、**「（様式第9号）重篤な有害事象に関する報告書」^*^**を用いる。

^*^臨床研究に関する様式ダウンロード<http://www.med.tohoku.ac.jp/public/rinri_d.html>

1. 多施設共同研究の場合、研究機関の研究責任者は、重篤な有害事象／不具合の発現を知った時点から以下の期限内に研究責任者（研究代表者）に報告する。報告は、**「（参考書式１）重篤な有害事象に関する報告書」**を用いる。
2. 他機関が研究代表施設の場合、対応は研究代表施設の手順に従う。

**研究機関の長、研究責任者**（研究代表者）への報告要否と報告期限

|  | | **軽症/中等症/重症（Grade1/2/3）** | | | | | | **最重症（Grade4）** | | **死亡** | | | **その他**  **医学的に**  **重要な**  **状態** |
| --- | --- | --- | --- | --- | --- | --- | --- | --- | --- | --- | --- | --- | --- |
|  | | **予測できる**  **（既知）** | | **予測できない**  **（未知）** | | | | **予測できる**  **（既知）** | **予測できない**  **（未知）** | **予測できる**  **（既知）** | **予測できない**  **（未知）** | |
|  | | **入院**  **なし／あり** | | **入院**  **なし** | | **入院**  **あり** | |
| **因果関係あり** | 報告  不要 | | 報告  不要 | | 初回報告  ：10日以内  追加報告  ：随時 | | 一次報告：72時間以内  二次報告：7日以内  追加報告：随時 | | | | |  | |
| **因果関係なし** | 報告  不要 | | 報告  不要 | | 初回報告  ：10日以内^*^  追加報告  ：随時^*^ | | 一次報告：72時間以内^*^  二次報告：7日以内^*^  追加報告：随時^*^ | | | | |  | |

^*^治療中または最終プロトコール治療日から30日以内のみ

# 共同研究機関への報告

1. 多施設共同研究の場合、研究責任者は、有害事象／不具合が発現した研究機関の研究責任者、共同研究機関の研究責任者に重篤な有害事象に関する報告書を用いて報告し、研究機関の長、倫理委員会への報告を依頼する。
2. 研究機関の研究責任者は、研究機関の長の指示を受け、必要な措置を講じる。

# 評価項目・方法

# 主要評価項目

本研究の目的を急性増悪予防のための遠隔看護介入プログラムの有効性を検証することとしているため、急性増悪の状況を評価項目として設定する必要がある。急性増悪は臨時受診回数や再入院回数を別々に考えただけでは判断できないため、本研究の主要評価項目は、過去12・24週間の急性増悪による臨時外来受診状況（受診率、回数）と再入院状況（入院率、回数）、各理由を参加登録時（ベースライン）、参加登録から12週後、参加登録から24週後に患者本人から収集する。患者本人の訴えが不明確な場合のみ、診療録より収集する。

# 副次的評価項目

副次評価項目は、呼吸器疾患の特異的な健康関連 QOL 評価尺度として開発されたSt. George’s Respiratory Questionnaire（以下SGRQ）、医療技術の経済評価において質調整生存年（Quality-Adjusted Life Year：以下QALY）の算出に用いるためのQOL評価尺度であるEuro Qol 5 Dimension（以下EQ-5D）、患者のセルフケアの程度を示す尺度であるセルフケア能力質問紙（Self-Care Agency Questionnaire：以下SCAQ）、呼吸機能検査による測定値、6分間歩行試験（6-minute walk test：以下6MWT）の距離を参加登録時（ベースライン）、参加登録から12週後と24週後に収集する。また、身体活動量の客観的な尺度として、平均歩数と活動量を参加登録から12週後と24週後に活動量計から収集する。

# 評価の中央判定

本研究では評価の中央判定は行わない。

# 登録・割付

# 登録

1. **登録の手順**

東北大学病院呼吸器内科および各協力機関の主治医が対象を選択し、定期外来時に調査説明書を配布する。調査説明書を読み、調査に関心を持った対象が自由意思により、研究者に初回面接の問い合わせを行う。初回面接では、適格基準の確認と調査説明を口頭および文書で行い、調査参加の同意書を提出した時点で本調査対象として登録する。

1. **登録に際しての注意事項**

　登録者が重複しないように、登録者リストを作成して管理する。また、研究開始後に必要な対象者数が集まっていないことが予想されるため、順次、登録する方法で対象者を加える。

# 割付

**割付方法、割付調整因子**

事前に調査予定人数について、乱数によって無作為に介入群および対照群へ割り付けた表を作成する。研究協力の同意が得られた対象に通し番号を付け、連結匿名化を行うこととする。研究補助者が通し番号から作成した乱数表に当てはめて、介入群および対照群に割り付ける。割付調整因子は多施設による調査となるので、施設によって異なる医療体制と考えられるため、施設ごとに割付を行う必要があると考えられる。

# 予定症例数、設定根拠

# 予定症例数

介入群と対照群の予定症例数をそれぞれ17名とし、総計34名の予定症例数とする。

# 設定根拠

本研究の主たる仮説は「遠隔看護介入プログラムを行うNPPVを受けている慢性呼吸不全患者群（介入群）は、介入期間の急性増悪による臨時外来受診（受診割合、回数）が通常の診療のみの対照群と比較して少ない。」とする。先行文献^24)^より、急性増悪によって救急外来を受診したNPPV実施者の平均回数は2.4回/年、NPPV非実施者の受診回数1.6回/年を参考に1.0回/年の改善をはかると仮定した。有意水準5％、検出力80％、改善すると仮定した受診回数の標準偏差を1.0回/年で算出して、介入群・対照群を各15名とする。また、研究協力の同意をしていても研究途中での脱落者の割合を全体の10％と見込んで算定を行うと介入群・対照群を各17名の必要症例数として、両群計34名の予定登録者数とした。

# 統計解析

# 統計解析の方法

対象の基本属性はKolmogorov-Smirnov testによる正規性の検討を行った後、正規性の有無によりmean t-testまたはmedian-testにより介入群と対照群の群間差を比較検討する。主要評価項目である臨時外来受診率と再入院率の群間比較はFisher's exact test、臨時受診と再入院の頻度の群間比較はStudent t-testを用いる。副次評価項目の変化は、介入開始前・参加登録時（ベースライン）、介入開始・参加登録から12週後、介入開始・参加登録から24週後（介入期間終了時）においてpaired t-testを用いる。なお、分析にはマイクロソフト社OS Windows8.1をインストールしたパソコン、統計ソフトウェアIBM SPSS Statistics Ver.21.0を用い、両側検定で有意水準は5％とする。

# 中間解析と研究の早期中止

介入開始・参加登録から12週後に中間解析を行い、主目的が達成されているか判断する。解析方法は本解析と同様の方法を用いる。また、中間解析の結果、研究対象者数が予定症例数に達しない場合でも介入群の優越性が十分であると認められた場合は研究の早期中止を行う場合がある。また、本介入に関連した有害事象が全体の10％以上の対象者に出現した場合は調査を中止する。

# データの管理方法、自己点検の方法

# 症例記録（Case Report Form：CRF）の作成

CRFの記載の記入及び訂正は研究者が行う。研究者は各被験者への介入が終了後、速やかにCRFを作成する。本プログラムは24週に及ぶため、CRF作成は介入24週後とする。主要評価項目や副次評価項目が網羅され、対象の状態が日々、どのように経過したのかバイタルサインなどが簡潔明瞭に把握できるように留意する。また、看護記録と同様に、テレビ電話等での情報提供が行われた際には内容がわかるように記録に残すこととする。

# CRFの自己点検

1. 研究分担者は、CRF内容と原資料（診療録、生データ等）の整合を確認する。
2. CRFと原資料に矛盾がある場合、その理由を説明する記録を作成する。
3. 研究機関の研究責任者または研究分担者は、作成されたCRFについてその内容を点検し、確認した上で記名・押印又は署名を行う。
4. 研究分担者がCRFの保管を行い、第三者の目に触れることのないように保管する。

# CRFの送付及び保管

研究機関の研究責任者は、作成したCRFを定められた手順「CRF記入の手引き」にて原本または複写をデータセンターに提出し、写しを保管する。提出先は下記とする。

**（CRFの提出先）**

東北大学病院臨床試験データセンター

住所：〒980-8574宮城県仙台市青葉区星陵町1番1号

[TEL：022-717-7122](tel:022-717-7137)

　CRF送付に関するデータセンター等の連絡は、研究対象者登録番号を用いる。CRFのFAX送信は行わず、帳票出力し紙媒体で提出するか電子申請によるCRFの登録を行う。紙媒体の場合、記載済CRFのコピーまたは紙媒体をスキャニングした電子媒体を研究機関の研究責任者が保管する。

# CRFの修正手順

CRFを訂正する場合、研究機関の研究責任者はCRFの変更又は修正の記録を定められた手順「CRF記入の手引き」にて作成・提出し、その写しを保管する。

# インフォームド・コンセントを受ける手続

# 研究対象者への説明

研究者は登録前に研究機関の承認を得た説明文書を研究対象者に渡し、以下の内容を説明する。本研究に該当がない項目については、説明文書内に該当なしを明記する。

**（説明文書記載事項）**

①研究の名称、研究実施について研究機関の長の許可を受けている旨

②研究機関、研究責任者

③研究の目的、意義

④研究の方法、期間

⑤研究対象者として選定された理由

⑥研究対象者に生じる負担並びに予測されるリスク、利益

⑦研究実施・継続に同意した場合も随時これを撤回できる旨

⑧研究実施・継続の不同意・同意撤回により研究対象者等が不利益な取扱いを受けない旨

⑨研究に関する情報公開の方法

⑩研究対象者等の求めに応じ他の研究対象者の個人情報等の保護や研究の独創性の確保に支障がない範囲内で研究計画書、研究の方法に関する資料入手・閲覧方法

⑪個人情報等の取扱い（匿名化する場合はその方法を含む）

⑫試料・情報の保存、廃棄の方法

⑬利益相反に関する状況（研究の資金源、起こり得る利害の衝突、研究者等の関連組織との関わり）

⑭研究対象者等及びその関係者からの相談等への対応

⑮研究対象者等に経済的負担・謝礼がある場合の内容

⑯侵襲を伴う研究の場合、研究によって生じた健康被害に対する補償の有無、内容

⑰研究対象者から取得された試料・情報について、研究対象者等から同意を受ける時点では特定されない将来の研究のために用いられる可能性または他の研究機関に提供する可能性がある場合の同意を受ける時点において想定される内容

# 同意

研究についての説明を行い、十分に考える時間を与え、研究対象者が試験の内容をよく理解したことを確認した上で、試験への参加について依頼する。研究対象者本人が試験参加に同意した場合、同意文書に研究対象者本人による署名を得る。同意文書は、1部を研究機関の研究責任者が保管し、1部を研究対象者本人に渡す。

# 代筆による同意取得の手続き

対象者に認知機能障害が生じておらず、身体的な障害等により筆記が困難な場合のみ、代筆により同意を得ることとする。代筆者は同居者とし、対象者と意思疎通が十分にはかれる者とする。代筆者による同意取得の場合の説明、同意に関する事項は対象者と同様とする。代筆を行った場合、対象者本人の同意について別途記録する。

# 個人情報等の取扱い

# 個人情報の利用目的

研究の正しい結果を得るために、治療中だけではなく治療終了後も長期間にわたり研究対象者個人を特定して調査を行うこと、取得した情報を適切に管理することを目的として個人情報を利用する。

# 利用方法（匿名化の方法）

研究対象者の個人情報保護のために連結可能匿名化を行い、連結匿名化のリストはUSBと共に鍵のついた棚で保管する。私は研究対象者IDを利用し、これ以外の個人情報は研究機関からデータセンターに開示しない。診療録番号は研究対象者IDに変換し、対応表により管理する。

# 安全管理責任体制（個人情報の安全管理措置）

研究機関の研究責任者は、個人情報利用にあたり安全管理対策を講じ情報流出リスクを最小化する。対象者データが携帯電話通信網上で安全に送受信されるよう管理するために、プライバシーマークの付与されたシステム開発会社にサーバーを設置し、データの取得、保管、廃棄を行う。本プログラムで使用するNTTドコモ東北の情報通信技術は、すでに遠隔的に高齢者生活の見守り活動を地域住民対象に行っており、独自のセキュリティシステムを構築しているため、情報漏洩の危険が少ないと考えた。NTTドコモへの情報提供は想定していない。データ通信時には仮想通信回線（Virtual Private Network:以下VPN）接続を行い、第三者からのデータ漏洩や改竄が不可能な状態に設定する。また、介入時は東北大学大学院医学系研究科がん看護学分野大学院生室等のプライバシーが保たれる個室で行い、データの閲覧、電話・テレビ電話中は研究者以外の入室を制限する。介入時の対応内容は速やかに主治医に報告し、主治医には対象者データの閲覧権限を付与、連携をもちながら実施する。得られた対象者データは大学院生室内で取り扱うこととする。データをUSBに保存する際はセキュリティロックを実施し、USB本体は鍵のついた棚に保管する。

# 研究対象者に生じる負担、予測されるリスク（起こりうる有害事象を含む）・利益、これらの総合的評価、負担・リスクを最小化する対策

# 研究参加に伴って予測される利益と不利益の要約

1. **予測される利益**

本研究で用いる遠隔看護介入プログラムは対象者に対して適応が考えられる看護支援方法であり、介入後の健康状態の改善が見込まれる。また、評価項目の呼吸機能検査や6分間歩行試験は日常診療外での検査ではあるが、研究者自身で機材等を準備し、検査するため、対象者に経済的な負担は伴わない。また、日常診療に比して、研究対象者が本研究に参加することで得られる特別な診療上、経済上の利益はない。

1. **予測される危険と不利益**

本研究で用いる遠隔看護介入プログラムは非侵襲的な介入であり、介入に関連した有害事象は少ないと考えられる。本研究に参加することで予測される不利益は患者の状態変化時の受診の遅れが挙げられる。本プログラムは全身状態が安定している場合を想定しているため、タブレット端末には急変時の通報機能を準備していない。そのため、状態が急変時には速やかな救急車の依頼、または対象者自身で病院を受診することとなる。その旨を対象者及びその家族が理解されるまで十分に説明し同意を得る。また、状態変化が明らかな場合は速やかに主治医または担当医に相談し、対処を依頼することとする。

# 試料・情報等の保存・廃棄の方法

# 保存

研究責任者は、試料・情報等を以下の通り保存する。

| **保存者** | **保存する試料・情報等** | **保存期間** |
| --- | --- | --- |
| **研究責任者** | ○研究機関において保存すべき研究に係る文書または原資料  ・患者背景となる基本情報  ・日々のバイタルサインデータ  ・質問紙調査項目  ・呼吸機能検査測定値、6分間歩行試験測定値  ○手順書　等 | 研究終了後5年 |

# 廃棄

研究責任者は、人体から取得した試料・情報等を廃棄する場合、匿名化する。

# 研究の資金源等、研究機関の研究に係る利益相反及び個人の収益等、研究者等の研究に係る利益相反に関する状況

本研究は文部科学省 科学研究費 若手研究(B)「慢性呼吸不全患者への身体活動の可視化を活用した新たな遠隔看護支援プログラムの構築」（研究課題番号16K20757）、電気通信普及財団研究調査助成金で実施される。本研究において企業との利益相反に値する関係はない。

# 知的財産

本研究により得られた結果やデータ、知的財産権は、東北大学、研究責任者に帰属する。具体的な取扱いや配分は協議して決定する。研究責任者の知的財産の帰属先を個人とするか研究機関とするかは、所属研究機関の取り決めに従う。

# 研究に関する情報公開の方法

# 研究計画の登録

研究責任者は公開データベースである大学病院医療情報ネットワーク（UMIN）http://www.umin.ac.jp/ctr/index-j.htmに研究概要を登録し、研究計画書変更、研究進捗に応じて適宜更新する。

# 研究結果の登録

研究責任者は、公開データベース等に研究終了後に研究結果を登録する。ただし、研究対象者等の人権、研究者等の関係者の人権、知的財産保護のため非公開とする事項、個人情報保護の観点から研究に著しく支障が生じるため倫理委員会の意見を受け研究機関の長が許可した事項は非公開とする。

# 研究結果の公表

研究責任者は、研究終了後、研究対象者の個人情報保護に措置を講じた上で、遅滞なく研究結果を医学雑誌等に公表する。結果の最終公表を行った場合、遅滞なく研究機関の長に報告する。

# 研究機関の長への報告内容、方法

　研究責任者は、以下を研究機関の長に**「（様式第8号）研究の進捗状況等に関する報告書」**により報告する。

・研究の進捗状況

・研究の実施に伴う有害事象の発生状況

・研究終了／中止、結果の概要

# 研究対象者等、その関係者からの相談等への対応

　研究全般に関する問合せ窓口、プライバシーポリシーに関する問合せ窓口

研究事務局、研究分担者

博士後期課程　霜山 真

東北大学大学院医学系研究科保健学専攻家族支援看護学講座がん看護学分野

TEL　090−7798−1240

E-mail　makoto.shimoyama.r7@dc.tohoku.ac.jp

# 研究対象者等に経済的負担または謝礼がある場合、その旨、その内容

研究対象者への予測される経済的負担は、タブレット端末を用いるため、その際に生じる充電代が生じるため、対象者に負担していただく。電池容量1000mAhで充電効率100％として、電気料金を1kWhあたり24円で計算すると、1回の充電におよそ0.88円となる。充電回数を多く見積もって1日1回とすると0.88円×180日で約160円となる。この自己負担についても対象者に十分に説明し、同意を得る。研究後の謝礼に関しては特に想定していない。

# 侵襲を伴う研究の場合、研究によって生じた健康被害に対する補償の有無、内容

研究の実施に起因して研究対象者に健康被害が発生した場合、研究機関は治療その他必要な措置を講じる。研究対象者の健康状態が悪化した際には速やかに受診を勧め、主治医または担当医から適切な治療が受けられるように配慮する。医療費の自己負担分については研究対象者の負担とする。医療費の自己負担分については研究対象者の負担とする。

# 試料・情報が同意を受ける時点では特定されない将来の研究のために用いられる可能性／他の研究機関に提供する可能性がある場合、その旨と同意を受ける時点において想定される内容

本研究で得られたデータは、本研究以外で二次利用することは想定していない。現時点では付随研究は計画されていないが、将来的に付随研究が計画された場合には研究計画書を新たに作成し、倫理委員会の審査を再び経た上で研究を実施する。

# 本研究の共同研究機関、あるいはそれ以外の研究機関への試料・情報等の提供

本研究で得られた試料・情報等を他の研究機関に提供することは想定していない。

# 営利団体等への試料・情報等の提供

本研究で得られた試料・情報等を他の営利団体に提供することは想定していない。

# 研究計画書の変更

研究計画書を変更する場合、研究責任者は、倫理委員会の審査を経て研究機関の長の承認を得る。

研究計画書内容の変更を、改正・改訂の2種類に分けて取扱う。その他、研究計画書の変更に該当しない補足説明の追加をメモランダムとして区別する。

1. **改正（Amendment）**

研究対象者の危険を増大させる可能性のある、または主要評価項目に影響を及ぼす研究計画書の変更。各研究機関の承認を要する。以下の場合が該当する。

①被験者に対する負担を増大させる変更（採血、検査等の侵襲の増加）

②重篤な副作用の発現による除外基準等の変更

③有効性・安全性の評価方法の変更

④症例数の変更

1. **改訂（Revision）**

研究対象者の危険を増大させる可能性がなく、かつ主要評価項目に影響を及ぼさない研究計画書の変更。各研究機関の承認を要する。以下の場合が該当する。

①被験者に対する負担を増大させない変更（検査時期の変更）

②研究期間の変更

③研究者の変更

1. **メモランダム／覚え書き（Memorandum）**

研究計画書内容の変更ではなく、文面の解釈上のバラツキを減らす、特に注意を喚起する等の目的で、研究責任者から研究関係者に配布する研究計画書の補足説明。

# 研究の実施体制

# 研究機関の名称、研究責任者の氏名

研究機関：東北大学大学院医学系研究科保健学専攻家族支援看護学講座がん看護学分野

研究責任者：教授　佐藤 冨美子

TEL　022−717−7926 FAX　022−717−7910

E-mail　fsato@med.tohoku.ac.jp

# 共同研究機関

1. 共同研究機関：あり

○機関名を特定できる場合

将来参加が予測される共同研究機関：東北大学病院および東北大学病院呼吸器内科から対象者を紹介している総合病院呼吸器内科とする。

| 機関名 | 研究責任者 | 倫理委員会の有無 |
| --- | --- | --- |
| 東北大学病院 | 呼吸器内科　小川浩正 医師 | 有 |
| 東北医科薬科大学若林病院 | 呼吸器内科　高橋識至 医師 | 有 |
| 石巻赤十字病院 | 呼吸器内科　小林誠一 医師 | 有 |
| 東北労災病院 | 呼吸器内科　田代祐介 医師 | 有 |
| 仙台医療センター | 呼吸器内科　三木 祐 医師 | 有 |
| みやぎ県南中核病院 | 呼吸器内科　岡田信司 医師 | 有 |
| 大崎市民病院 | 呼吸器内科　井草龍太郎 医師 | 有 |

○当該研究機関に倫理委員会がある場合

東北大学大学院医学系研究科倫理委員会を承認後、速やかに研究責任者となる医師に連絡し、各協力施設の倫理委員会の承認を得ることとする。

○当該研究機関に倫理委員会がない場合

倫理委員会がないことに対する対処法：研究機関から東北大学大学院医学系研究科倫理委員会に審査を依頼し、承認を得る。

1. 共同研究機関の役割：対象者の紹介、有害事象時の対応
2. 試料・情報の収集に際し匿名化を行う

（理由：対象施設において患者の個人情報保護のために　連結可能匿名化を行うこととする。）

1. 匿名化を行う場合

匿名化を行う機関：試料・情報を収集した共同機関と当院

（理由：対象施設において患者の個人情報保護のために　連結可能匿名化を行うこととする。）

1. 他施設でのインフォームド・コンセントの内容を確認した

（理由：他施設におけるインフォームド・コンセントはすべて研究分担者が行うため、内容の確認は済んでいる。）

1. 共同研究機関へ試料・情報を提供しない

# 研究事務局、研究者の役割

1. 研究事務局、試験機器管理者：

博士後期課程　霜山 真

東北大学大学院医学系研究科保健学専攻家族支援看護学講座がん看護学分野

TEL　090−7798−1240

E-mail　makoto.shimoyama.r7@dc.tohoku.ac.jp

1. 研究分担者：

博士後期課程　霜山 真

東北大学大学院医学系研究科保健学専攻家族支援看護学講座がん看護学分野

TEL　090−7798−1240

E-mail　makoto.shimoyama.r7@dc.tohoku.ac.jp

准教授　小川浩正

東北大学病院呼吸器内科／東北大学大学院医学系研究科産業医学分野

TEL　 022-717-7874

E-mail　ogawa-hiro@m.tohoku.ac.jp

助教　佐藤輝幸

東北大学大学院医学系研究科内科病態学講座 呼吸器内科学分野

TEL　 022-717-8539

E-mail　satoteruyuki@gmail.com

1. 試料・情報等の保存・管理責任者：

教授　佐藤 冨美子

東北大学大学院医学系研究科保健学専攻家族支援看護学講座がん看護学分野

TEL　022−717−7926 FAX　022−717−7910

E-mail　fsato@med.tohoku.ac.jp

# 統計解析、データセンター

1. 統計解析責任者、データ管理者：

教授　佐藤 冨美子

東北大学大学院医学系研究科保健学専攻家族支援看護学講座がん看護学分野

TEL　022−717−7926 FAX　022−717−7910

E-mail　fsato@med.tohoku.ac.jp

1. データマネジメント従事者：

博士後期課程　霜山 真

東北大学大学院医学系研究科保健学専攻家族支援看護学講座がん看護学分野

TEL　090−7798−1240

E-mail　makoto.shimoyama.r7@dc.tohoku.ac.jp

# 研究に関する問合せ窓口

研究対象者の登録方法、有害事象発生時の対応方法：

博士後期課程　霜山 真

東北大学大学院医学系研究科保健学専攻家族支援看護学講座がん看護学分野

TEL　090−7798−1240

E-mail　makoto.shimoyama.r7@dc.tohoku.ac.jp

# 引用文献

1) 厚生労働省（2016），平成28年版厚生労働白書－人口高齢化を乗り越える社会モデルを考える－，2016年12月5日閲覧，http://www.mhlw.go.jp/wp/hakusyo/kousei/16/dl/all.pdf．

2) 文部科学省（2012），長寿社会における生涯学習の在り方について～人生100年いくつになっても学ぶ幸せ「幸齢社会」～，2016年12月5日閲覧，http://www.mext.go.jp/component/a_menu/

education/detail/__icsFiles/afieldfile/2012/03/28/1319112_1.pdf．

3) 「高齢者に対する適切な医療提供に関する研究」研究班：高齢者に対する適切な医療提供の指針，日本老年医学会誌，51，p.89-96，2014．

4) 社会保障制度改革推進本部（2015），医療・介護情報の活用による改革の推進に関する専門調査会第１次報告～医療機能別病床数の推計及び地域医療構想の策定に当たって～，2016年12月5日閲覧，http://www.kantei.go.jp/jp/singi/shakaihoshoukaikaku/houkokusyo1.pdf．

5) 社会保障制度改革国民会議（2013），社会保障制度改革国民会議報告書～確かな社会保障を将来世代に伝えるための道筋～，2016年12月5日閲覧，http://www.kantei.go.jp/jp/singi/kokuminkaigi/

pdf/houkokusyo.pdf．

6) 厚生労働省（2014），健康・医療・介護分野におけるICT化の推進について，2016年12月5日閲覧，http://www.mhlw.go.jp/file/06-Seisakujouhou-12600000-Seisakutoukatsukan/0000042495.

pdf．

7) 総務省（2013），ICT超高齢社会構想会議報告書－「スマートプラチナ社会」の実現－，2016年12月5日閲覧，http://www.soumu.go.jp/main_content/000268318.pdf．

8) Nici L，ZuWallack R，American Thoracic Society Subcommittee on Integrated Care of the COPD Patient(2012)：An official American Thoracic Society workshop report: the Integrated Care of The COPD Patient，Proc Am Thorac Soc，9(1)，p.9-18.

9) Spruit MA，Singh SJ，Garvey C，et al(2013)：An official American Thoracic Society/European Respiratory Society statement: key concepts and advances in pulmonary rehabilitation，Am J Respir Crit Care Med，188(8)，p.e13-64．

10) Adams SG，Smith PK，Allan PF，et al(2007)：Systematic review of the chronic care model in chronic obstructive pulmonary disease prevention and management，Arch Intern Med，167(6)，p.551-61.

11) Farida F, Anne M, Dominique L.C, et al(2014)：The effect of an outpatient care on-demandsystem on health status and costs in patients with COPD. A randomized trial, Respiratory Medicine (2014) 108, p.1163-1170.

12) Claire L B, Gail A M, Jill T, et al(2014)：A pilot randomised controlled trial of a Telehealth intervention in patients with chronic obstructive pulmonary disease: challenges of clinician-led data collection, Trials, 15, 313.

13) Shwamm LH, Holloway RG, Amarenco P, et al(2009)：A review of the evidence for the use of telemedicine within stroke systems of care：a scientific statement from the American Heart Association/American Stroke Association, Stroke 40, p.2616-2634.

14) Leonie T, Job van der H, Christian M, et al(2014)：Telepulmonology and telespirometry, e-Health For Continuity of Care, 205, p.211-215.

15) León A, Cáceres C, Fernández E, et al(2011)：A New Multidisciplinary Home Care Telemedicine System to Monitor Stable Chronic Human Immunodeficiency Virus-Infected Patients: A Randomized Study, PLoS One, 6(1), e14515.

16) 亀井 智子, 山本 由子, 梶井 文子ら(2011)：COPD在宅酸素療法実施者への在宅モニタリングに基づくテレナーシング実践の急性増悪および再入院予防効果　ランダム化比較試験による看護技術評価, 日本看護科学会誌, 31巻2号, p.24-33.

17) 東 ますみ(2012)：2型糖尿病患者に対する遠隔看護介入の自己管理行動への影響, 日本遠隔医療学会雑誌, 8巻2号, p.158-161.

18) 菊地 ひろみ, 照井 レナ, 柿山 浩一郎ら(2013)：広域寒冷積雪地における地域完結型遠隔看護システムの構築 在宅療養者に対する効果, 日本遠隔医療学会雑誌, 9巻2号, p.159-162.

19) FARIDA F.B, JAN W.K, STEVEN M.U, et al(2015)：Telemedicine, the effect of nurse-initiated telephone follow up, on health status and health-care utilization in COPD patients: A randomized trial, Respirology, 20, p.279-285.

20) 総務省情報流通行政局地域通信振興課(2011)：遠隔医療モデル参考書, 2016年12月5日閲覧, http://www.soumu.go.jp/main_content/000127781.pdf.

21) 日本呼吸器学会肺生理専門委員会・日本呼吸管理学会酸素療法ガイドライン作成委員会（2006）：酸素療法ガイドライン, メディカルビュー社.

22) WHO(2008）：World health statistics 2008.

23) 杉山幸比古, 門田淳一, 弦間昭彦(2016)：呼吸器疾患 最新の治療2016-2018, 南江堂.

24) 日本呼吸器学会在宅呼吸ケア白書作成委員会（2013）：在宅呼吸ケア白書 COPD患者アンケート調査疾患別解析, メディカルビュー社.

25) 日本呼吸器学会NPPVガイドライン作成委員会(2015)：NPPVガイドライン 改訂第2版,南江堂.

26) Spruit MA, Singh SJ, Garvey C, et.al. (2014)：An official American Thoracic Society/European Respiratory Society statement : key concepts and advances in pulmonary rehabilitation. Am J Respir Crit Care Med, 189(12), 1570.

27) 霜山真, 古瀬みどり(2012)：在宅非侵襲的陽圧換気療法を行っている慢性呼吸不全患者のセルフケア獲得プロセス, 日本看護研究学会誌, 35(2), 1-10.

28) 日本呼吸器学会COPDガイドライン第4版作成委員会(2013)：COPD診断と治療のためのガイドライン(第4版), メディカルレビュー社.

29) de Marco R, Accordini S, Marcon A, et al(2011)：Risk factors for chronic obstructive pulmonary disease in a European cohort of young adults, Am J Respir Crit Care Med, 183(7), 891-7.

30) 日本呼吸ケア・リハビリテーション学会呼吸リハビリテーション委員会 他（2007）：呼吸リハビリテーションマニュアル-患者教育の考え方と実践-, 照林社.

31) Hurst JR, Vestbo J, Anzueto A, et al(2010)：Susceptibility to exacerbation in chronic obstructive pulmonary disease, N Engl J Med, 363(12), 1128-38.

32) Samantha L.H, Tania J.F, Dina B, et al(2015)：Self-Management Following an Acute Exacerbation of COPD A Systematic Review, Chest, 147(3), 646-61.

33) Gosselink R, De Vos J, van den Heuvel SP, et al(2011)：Impact of inspiratory muscle training in patients with COPD: what is the evidence?, Eur Respir J, 37(2), 416-25.

34) Rice K, Bourbeau J, MacDonald R, et al(2014)：Collaborative self-management and behavioral change, 35(2), 337-51.

35) Jefferson T, Foxlee R, Del Mar C, et al(2008): Physical interventions to interrupt or reduce the spread of respiratory viruses: systematic review, BMJ, 336, 77-80.

36) Song HY, Yong SJ, Hur HK(2014)；Effectiveness of a brief self-care support intervention for pulmonary rehabilitation among the elderly patients with chronic obstructive pulmonary disease in Korea, 39(3), 147-56.

37) 三塚由佳, 高橋識至, 飯田聡美ら(2015)：COPD増悪時アクションプランにおける問題点と電話指導の効果. 日本呼吸ケア・リハビリテーション学会, 第25巻, 第1号.

38）Gadoury MA, Schwartzman K, Rouleau M, et al (2005)：Self-management reduces both short- and long-term hospitalisation in COPD, Eur Respir J, 26(5), 853-7.

39）Mendoza L, Horta P, Espinoza J, et al (2015) : Pedometers to enhance physical activity in COPD: a randomised controlled trial. Eur Respir J, 45(2), 347-54.

40) 植木純(2015)：呼吸リハビリテーションと身体活動性―身体活動性の向上・維持に関する現況と課題―, 日本呼吸器学会誌, 4巻1号, p.36-40.

41) Jones PW, Quirk FH, Baveystock CM.(1991)：The St George's Respiratory Questionnaire, Respir Med, 85 Suppl B, p.25-31.

42) Nishimura K, Tsukino M, Hajiro T.(1998)：Health-related quality of life in patients with chronic obstructive pulmonary disease, Curr Opin Pulm Med, 4(2), p.107-15.

43) TAKASHI HAJIRO, KOICHI NISHIMURA, MITSUHIRO TSUKINO, et al.(1998)：Comparison of Discriminative Properties among Disease-specific Questionnaires for Measuring Health-related Quality of Life in Patients with Chronic Obstructive Pulmonary Disease, Am J Respir Crit Care Med, Vol 157, p.785–790.

44) 西村正治，牧田比呂仁(2013)：北海道COPDコホート研究, 日本内科学会雑誌, 102(2), p.463-470.

# Appendix

・説明・同意文書

・症例報告書

・連結可能匿名化対応表

・質問紙表
