# Supplementary material for: Effectiveness of a telenursing intervention program in reducing exacerbations in patients with chronic respiratory failure receiving noninvasive positive pressure ventilation: A randomized controlled trial
Source: PLoS One. 2023 Oct 26;18(10):e0269753. doi: 10.1371/journal.pone.0269753 (PMC10602241; doi:10.1371/journal.pone.0269753)
Supplement: Attachment — Submitted filename: Response_to_Reviewers.docx [file pone.0269753.s009.docx]

November 7, 2022

Jamie Males

Executive Editor

*PLOS ONE*

Dear Jamie Males:

Thank you for inviting us to submit a revised draft of our manuscript entitled, “Effectiveness of a telenursing intervention program in reducing exacerbations in patients with chronic respiratory failure receiving noninvasive positive pressure ventilation: A randomized controlled trial” to *PLOS ONE*. We also appreciate the time and effort you and each of the reviewers have dedicated to providing insightful feedback on ways to strengthen our paper. Thus, it is with great pleasure that we resubmit our article for further consideration. We have incorporated changes that reflect the detailed suggestions you have graciously provided. We also hope that our edits and the responses we provide below satisfactorily address all the issues and concerns you and the reviewers have noted.

We hope that the revised manuscript is now suitable for publication in your journal.

Thank you for your consideration. We look forward to hearing from you.

Sincerely,

Makoto Shimoyama

School of Nursing

Miyagi University

1-1 Gakuen, Taiwa-cho, Kurokawa-gun, Miyagi, 981-3298, Japan

Telephone: +81 22 377 8277

To facilitate your review of our revisions, the following are point-by-point responses to the questions and comments delivered in your letter dated September 7, 2022

**JOURNAL REQUIREMENTS：**

1. Please ensure that your manuscript meets PLOS ONE's style requirements, including those for file naming.

**RESPONSE:** Thank you for your suggestion. We have reviewed and confirmed the style requirements again.

2. Thank you for submitting your clinical trial to PLOS ONE and for providing the name of the registry and the registration number. The information in the registry entry suggests that your trial was registered after patient recruitment began. PLOS ONE strongly encourages authors to register all trials before recruiting the first participant in a study.

**RESPONSE:** Thank you for your suggestion. We registered this study as a clinical trial with UMIN-CTR. We checked UMIN-CTR again and found that the clinical trial was registered on June 6, 2017. Recruitment of participants for the study began in September 2017, so registration was not delayed. For these reasons, no additional information has been added.

Please check the following URL;

<https://center6.umin.ac.jp/cgi-open-bin/ctr/ctr.cgi?function=brows&action=brows&recptno=R000031552&type=summary&language=J>

Please check the "Management information" in the web page provided. The "Registered date" was June 06, 2017. We initially thought that you wanted confirmation on “Date of disclosure of the study information,” which was June 20, 2019; however, this was not the registration date.

**Reviewer #1: PONE-D-22-13518: statistical review**

**Reviewers' comments:**

**MAJOR ISSUES**

1. The outcomes of the treatment and the control group are separately compared by two independent batteries of Mann-Whitney tests. This approach ignores the longitudinal structure of the data: individuals are observed at two different occasions. I would suggest performing a single battery of tests on the temporal differences of the outcomes. Specifically, the authors should first compute the individual difference between the value of each outcome at the end of the follow-up and the value at baseline. They should then perform a test that compares the differences in the treatment group with the differences in the control group.

**RESPONSE:** Thank you for your suggestion. The details of the analysis were omitted to simplify the presentation of the results. The data has been re-analyzed, and Table 3 has been modified to show the comparison test of the difference between the intervention and target groups.

The research methodology and results have been revised as follows:

1. Research Methods

・Page 11, lines 187–190: "For baseline values, the endpoints in the intervention and control groups were compared at the time of enrollment. The differences between the endpoints at baseline and 3 months after enrollment were then calculated, and the differences were used to compare the intervention and control groups. Mann-Whitney U test was used for comparison."

2. Results

・Table 3 has been modified to compare the differences between values at baseline and at 3 months after enrollment.

・The description of Table 3 within the results has been modified.

2. Table 2 seems to indicate that the primary outcomes (Number of hospitalizations in the past 3 months, Number of days hospitalized in the past 3 months, Number of unscheduled outpatient visits in the past 3 months) do not vary much within each sample. How is this possible? These data need some clarification. In addition, could the author please provide the single numbers? See also specific point 4.

**RESPONSE:** Thank you for your suggestion. Per your observation, the primary outcome data were not clear. We have summarized the values of the primary outcome for the participants in each group in tables and provided them as Supplementary Files 1-3.

**Specific points**

1. Tables 2 and 3: the total scores of SGRQ and SCAQ are not the sums of the partial scores. Please clarify.

**RESPONSE:** Thank you for your suggestion. Tables 2 and 3 show the median of the total and partial scores of the SGRQ and SCAQ, not necessarily the sum of the partial scores. Accordingly, it is now indicated in the table that it is not the sum of the partial scores, because of the median of the SGRQ and SCAQ.

2. Lines 252-268 are just a description of Table 2 and could be shortened,

**RESPONSE:** Thank you for your suggestion. The content has been condensed per your recommendation.

3. Lines 120-122: "Research assistants ... numbers". This sentence is unnecessarily repeated on line 127.

**RESPONSE:** Thank you for your observation, with which we agree. Accordingly, the redundant content has been removed.

4. Data are not provided. Data should be provided as a supplementary file or made available in a public repository.

**RESPONSE:** Thank you for your suggestion. To improve clarity regarding the primary outcome, a supplemental file of the data has been provided for your review.
