# Supplementary material for: Effectiveness of a telenursing intervention program in reducing exacerbations in patients with chronic respiratory failure receiving noninvasive positive pressure ventilation: A randomized controlled trial
Source: PLoS One. 2023 Oct 26;18(10):e0269753. doi: 10.1371/journal.pone.0269753 (PMC10602241; doi:10.1371/journal.pone.0269753)
Supplement: Attachment — Submitted filename: Response_to_Reviewers.docx [file pone.0269753.s010.docx]

February 4, 2023

Kathleen Finlayson

Academic Editor

*PLOS ONE*

Thank you for inviting us to submit a revised draft of our manuscript entitled, “**Effectiveness of a telenursing intervention program in reducing exacerbations in patients with chronic respiratory failure receiving noninvasive positive pressure ventilation: A randomized controlled trial**” to *PLOS ONE*. The manuscript ID is PONE-D-22-13518R1. We also appreciate the time and effort you and each reviewer have dedicated to providing insightful feedback on ways to strengthen our paper. Thus, it is with great pleasure that we resubmit our article for further consideration. We have incorporated changes that reflect the detailed suggestions you have graciously provided. We also hope that our edits and the responses we provided below satisfactorily address all the issues and concerns you and the reviewers have noted.

To facilitate your review of our revisions, the following is a point-by-point response to the questions and comments delivered in your letter dated 2022.12.2. We hope that the revised manuscript is now suitable for publication in your journal.

Thank you for your consideration. We look forward to hearing from you.

Sincerely,

Makoto Shimoyama,

School of Nursing,

Miyagi University,

1-1 Gakuen, Taiwa-cho, Kurokawa-gun, Miyagi, 981-3298, Japan.

Telephone: +81 22 377 8277

Additional Editor Comments;

1. In particular, the previous query on the need to allow for the longitudinal nature of the data suggests the need for a two-way data analysis, e.g. a Friedman test?

**RESPONSE:** We would like to thank the reviewer for this comment. In this analysis, the Mann–Whitney U test was used to calculate the corresponding amount of change that occurred before and after the intervention with the same participants and to compare the amount of change between the two groups (intervention and control). As there was no correspondence between the two groups, we do not think there is any problem with the method of analysis using the Mann–Whitney U test. As the reviewer suggested, the Friedman test is used for pre- and post-intervention comparisons; thus, we did not use it in this analysis.

2. I note in table 3 that there is no Mann-Whitney U test result/SE reported, which would be of interest.

**RESPONSE:** We would like to thank the reviewer for this comment. The Mann–Whitney U test is a nonparametric test. Thus, we used median and quartile deviations to denote it. We did not add the mean and standard errors because we believe they are used when parametric tests are used.

3. Another important omission is information on a sample size /power calculation - there are very few outcome events in your study, and as such, it appears underpowered. Providing this information would give a balanced view of your results.

**RESPONSE:** We would like to thank the reviewer for this comment. We did not make a statement regarding sample size/detection power, so we figured it was not clear. We have added a note regarding sample size/power calculations in the “Participants” subsection as follows:

“The average number of hospitalizations among NPPV practitioners in Japan was 2.0 times/year, and it was assumed that the average number of hospitalizations would improve by 1.0 time/year. The required sample size was calculated with a significance level of 5%, a power of 80%, and a standard deviation of the assumed average number of hospitalizations of 1.0 time/year. As a result, the intervention and control groups were set at 15 participants each.” (Lines 95–99)

4. On a minor note, giving the effect size results in the abstract besides wording which is talking about differences in outcomes is a little confusing, I’d just leave in the p value and note which test was done.

**RESPONSE:** We would like to thank the reviewer for this comment. We agree with the reviewer. The abstract was difficult to understand. We have excluded the effect size and only indicated the p-values. Moreover, we have stated that we used the Mann–Whitney U test for our analysis (Line 34).

5. Avoid including your findings in the introduction/aim section.

**RESPONSE:** We would like to thank the reviewer for this comment. We have reviewed the text and removed the discussion on the findings of our study that were included in the Introduction. Please check it.

6. Further information on the number of patients who fitted the inclusions criteria at recruitment sites, compared to the number who agreed to participate is needed - were all patients who fitted the criteria invited?

**RESPONSE:** We would like to thank the reviewer for this question. The number of patients who met the inclusion criteria at the recruiting facility was 33. We did not explain it well enough. We have added the following part to present this information clearer:

“In total, 33 patients met the participation criteria at the recruiting facility, all of whom were referred by the facility. Of the 33 participants referred by the facilities, 31 (n=15, intervention group; n=16, control group) agreed to participate in the study.” (Lines 198–200)

7. Many of the references are very old - updating is needed. As this is an international journal, only references which are available to an international audience should be included. National policy documents should thus have a URL or DOI.

**RESPONSE:** We would like to thank the reviewer for this comment. We have reviewed and updated our citations and references. We have also included the URL or DOI as necessary. Please review the references.
