# Supplementary material for: Effectiveness of a telenursing intervention program in reducing exacerbations in patients with chronic respiratory failure receiving noninvasive positive pressure ventilation: A randomized controlled trial
Source: PLoS One. 2023 Oct 26;18(10):e0269753. doi: 10.1371/journal.pone.0269753 (PMC10602241; doi:10.1371/journal.pone.0269753)
Supplement: Attachment — Submitted filename: Response_to_Reviewers.docx [file pone.0269753.s011.docx]

**Manuscript: PONE-D-22-13518R2**

**Response to Reviewers**

**Review Comments to the Author;**

**Reviewer #2:** This is a well-designed pragmatic randomized controlled trial that wanted to assess the telenursing intervention program for chronic respiratory failure receiving NPPV. However, some important issues were not clearly reported or conflicting.

**RESPONSE:** Thank you very much for your comments and for making us aware of the inconsistencies and problems in the manuscript. I sincerely appreciate it. I have corrected the manuscript in accordance with your comments.

1. How long does the telenursing intervention program last in the trial?

**RESPONSE:** Thank you very much for your query. The telenursing intervention program was intended to continue for 3 months. This point has been added to the "Telenursing intervention program" section.

2. Considering the sample size estimation, the authors assumed that the average number of hospitalizations would improve by 1.0 time per year. However, the primary outcome of the trial was the number of acute exacerbations in the past 3 months. It is clear that the sample size calculation is not supported by the expected data as the number of exacerbations in the past 3 months is likely less than within one year.

**RESPONSE:** Thank you very much for your comment. We acknowledge the discrepancy. Per your observation, we have corrected it to 0.25 improvement per 3 months instead of per year.

3. As for the statistical analysis, the ITT analysis principle, missing data processing, and co-variate and adjustment methods would be considered.

**RESPONSE:** Thank you very much for your comment. We added “In this study, the analysis took into account the principles of the ITT analysis principle, missing data processing, and co-variate and adjustment methods.” to the statistical analysis section.

4. Tests of baseline differences in the table 1 are unnecessary and illogical. Such hypothesis testing is superfluous and can mislead investigators and their readers. In addition, I would just leave in the statics and p value and note which test was done in the table 2 and 3.

**RESPONSE:** Thank you very much for your comment. We removed the baseline test from Table 1 and added that the Mann-Whitney U-test was performed; this was added in the section describing the contents of Tables 2 and 3.

Reviewer #3: This appears to be a well thought out and important study to address the needs of NPPV patients that require self-care support. The study is clear and logical and follows the CONSORT guidelines for reporting RCTs. The only comments that I have are minor.

**RESPONSE:** Thank you very much for your comments and for making us aware of inconsistencies and problems in the manuscript. I sincerely appreciate it. I have corrected the manuscript in accordance with your comments.

Page 7 Line 114 – Can the investigators please include a short description of the COMPAS system with particular reference to whether this system has an alert function that is sent to clinicians when signs of an acute exacerbation are detected. Please describe the responsibilities of the clinicians in the event that they are not notified. This is because in the Discussion on Page 20 Lines 285-287 ‘Some participants thought that they should not attempt outpatient visits outside of their regular appointments and chose to wait until the next regular outpatient visit, despite having symptoms of an acute exacerbation.’

**RESPONSE:** Thank you very much for your comment. We added a description of "COMPAS". Further, the alert function for clinicians was not included, therefore we have added that information as well. We always established a system for communication between researchers and clinicians, and never failed to notify clinicians. This explanation has also been added.

Page 9 Line 146 – Can the investigators please describe where the health information on the ‘server … managed by a system production and development company’ was stored and did this become part of the patient medical record managed by the hospital. Please also describe the implications of this in the discussion section.

**RESPONSE:** Thank you very much for your recommendation. The following information has been added to the discussion: " Additionally, due to security issues within the hospital, the patient health information obtained in this study was not integrated into the hospital's electronic medical record. We believe that this is one reason that, although various tele-nursing systems are currently being developed, their implementation has been hindered."

.

Page 9 Line 152 – Can the investigators please include a description of how the NFC function ‘was used to collect vital signs data’ and how it ‘assisted the participants with inputting data on vital signs.’

**RESPONSE:** Thank you for your recommendation. We added that the NFC functionality facilitated the automatic transfer of patient vital sign data from the measurement device to the tablet.

Page 10 Lines 165-167 – Can the investigators please include a description of what the SGRO scores represented and likewise for the SCAQ and QOL5 scores so that interpretation of data in the results section is facilitated.

**RESPONSE:** Thank you for your recommendation. We added explanations for each survey item scale.

Page 21 Line 301 – can the investigator please clarify what is meant by “clear respiratory symptoms” and what type of “sensations” are being referred to in the sentence:

“Signs of an acute exacerbation include not only the appearance of clear respiratory symptoms but also sensations that are somehow different from the norm”

**RESPONSE:** Thank you for your recommendation. We added explanations to the text regarding "clear respiratory symptoms" and "unusual sensations”.
